# A Path-Following Controller for Marine Vehicles Using a Two-Scale Inner-Outer Loop Approach

**DOI:** 10.3390/s22114293

**Published:** 2022-06-05

**Authors:** Pramod Maurya, Helio Mitio Morishita, Antonio Pascoal, A. Pedro Aguiar

**Affiliations:** 1 CSIR-National Institute of Oceanography, Dona Paula 403004, Goa, India; 2 Department of Naval Architecture and Ocean Engineering, Polytechnic School, University of São Paulo, São Paulo Campus, São Paulo 05508-030, Brazil; hmmorish@usp.br; 3 Institute for Systems and Robotics (ISR), IST, University of Lisbon, 1049-001 Lisbon, Portugal; antonio@isr.tecnico.ulisboa.pt; 4 Research Center for Systems and Technologies and Department of Electrical and Computer Engineering, Faculty of Engineering, University of Porto, 4200-465 Porto, Portugal; pedro.aguiar@fe.up.pt

**Keywords:** path following, inner-outer loop control, input-to-output stability, AUVs, ASVs

## Abstract

This article addresses the problem of path following of marine vehicles along straight lines in the presence of currents by resorting to an inner-outer control loop strategy, with due account for the presence of currents. The inner-outer loop control structures exhibit a fast-slow temporal scale separation that yields simple “rules of thumb” for controller tuning. Stated intuitively, the inner-loop dynamics should be much faster than those of the outer loop. Conceptually, the procedure described has three key advantages: (i) it decouples the design of the inner and outer control loops, (ii) the structure of the outer-loop controller does not require exact knowledge of the vehicle dynamics, and (iii) it provides practitioners a very convenient method to effectively implement path-following controllers on a wide range of vehicles. The path-following controller discussed in this article is designed at the kinematic outer loop that commands the inner loop with the desired heading angles while the vehicle moves at an approximately constant speed. The key underlying idea is to provide a seamless implementation of path-following control algorithms on heterogeneous vehicles, which are often equipped with heading autopilots. To this end, we assume that the heading control system is characterized in terms of an IOS-like relationship without detailed knowledge of vehicle dynamics parameters. This paper quantitatively evaluates the combined inner-outer loop to obtain a relationship for assessing the combined system’s stability. The methods used are based on nonlinear control theory, wherein the cascade and feedback systems of interest are characterized in terms of their IOS properties. We use the IOS small-gain theorem to obtain quantitative relationships for controller tuning that are applicable to a broad range of marine vehicles. Tests with AUVs and one ASV in real-life conditions have shown the efficacy of the path-following control structure developed.

## 1. Introduction

The use of autonomous marine vehicles, including surface and underwater robots, for various scientific and commercial applications at sea, has increased multi-fold in the last decade. Missions of interest include, among others, bathymetric surveys, seabed imaging, environmental monitoring, inspection of offshore critical infrastructures, and marine archaeology studies. In most of these missions, marine vehicles are required to follow spatial paths accurately. A representative example is the case where an AUV (autonomous underwater vehicle) or an ASV (autonomous surface vehicle) is requested to execute “lawn-mowing” maneuvers along desired paths in the presence of unknown ocean currents.

We recall the crucial difference between trajectory tracking and path following. In the latter, no explicit temporal constraints are imposed on the desired vehicle’s motion and a path is planned using spatial coordinates only, along with a reference speed profile that may depend on where the vehicle is on the path. In contrast, in trajectory tracking, space and time explicitly define the reference coordinates for the desired vehicle’s motion, that is, the vehicle is required to track a 3D curve parameterized in time. This strategy is only pursued in practice when simultaneous and temporal specifications play a decisive role. However, in the process of tracking a desired inertia trajectory, a vehicle may be required to reach a speed with respect to the water that may either be too small, leading to the loss of surface control authority or too high, exceeding the capability of the propulsion system installed onboard.

A properly designed path-following control systems can naturally lead to smoother vehicle trajectories without pushing the control signals into saturation, in contrast to what may happen when trajectory tracking controllers are used. The fundamental limitations of trajectory tracking can be found in [1,2]. In [2], Aguiar studied performance limitations of trajectory tracking strategies due to unstable zero-dynamics in terms of a lower bound on $L_{2}$-norm of the tracking error even if the control effort is unlimited. It was also shown that path following is free from such a limitation. In [3], a hybrid solution to path following and trajectory tracking problem for underactuated vehicles was discussed for 2D and a more general 3D space. Aguiar and Hespanha in [3] followed a supervisory control architecture in which a switching logic is used to adapt an adequate estimator and control law from the family of estimators and candidate control laws. Supervisory control combined with a nonlinear Lyapunov-based tracking control was demonstrated and its robustness to parametric modeling uncertainties was shown with examples of a hovercraft and an AUV. Marine vehicles suffer from disturbances induced by ocean currents. The issue of disturbances was not addressed in [3]. The path following problem in 3D for an underwater vehicle was also described in [4], where the controller design builds on the Lyapunov theory and resorts to back-stepping techniques, demanding the knowledge of a complete hydrodynamic model of the vehicle.

In [5], Indiveri described a 3D kinematical solution to path following by recalling sliding-mode control techniques. Rather than designing the control input to drive a tracking error to zero, the sliding mode control uses the control input to drive and keep the state on a surface where the error has stable dynamics. However, the authors did not address the issues related to the vehicle dynamics and described the controller only at the kinematic level.

Pioneering work in solving the path following problem for wheeled robots has been addressed in [6,7]. Path-following problem for a car pulling several trailers is addressed in [8]. In [9], Altafini provided local asymptotic stability for a path of non-constant curvature for a trailer vehicle. More recently, in [10], a model predictive path-following control of a laboratory tower crane has been described to move a load along a predefined geometric path.

**Figure 1:**
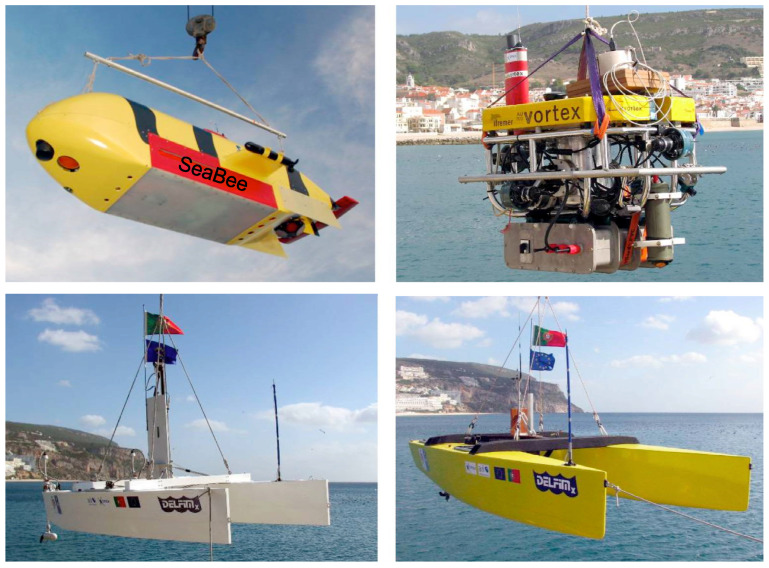
Heterogeneous vehicle used to demonstrate cooperative motion control during GREX trails at Sesimbra, Portugal.

It is interesting to see in [11,12] that path following is at the core of cooperative motion control for multiple vehicles where these vehicles are supposed to follow a set of fixed spatial paths while holding a desired formation pattern. Each vehicle is equipped with a path-following algorithm to maneuver along its assigned spatial path, whereas a distributed control law performs the formation control by adjusting the speeds of different vehicles. It is therefore important to emphasize the need for a reliable path-following method that is suitable for heterogeneous vehicles with little knowledge of their dynamics. Cooperation among multiple vehicles with a view to performing different tasks plays a critical role in executing a number of mission scenarios [13]. The GREX project is an example of the use of cooperative-motion control strategies involving a number of vehicles developed by different oceanographic institutions for their needs. Figure 1 illustrates the diversity in the marine vehicles used for cooperation during the sea trials of the GREX project.

The EU-funded project MORPH (FP7-ICT-2011-7 GA 288704, 2012–2016) [14] advanced the novel concept of an underwater robotic system composed of a number of spatially separated mobile robot-modules, carrying distinct and yet complementary resources with path-following algorithms implemented on every vehicle. MORPH provided the foundation for efficient methods to survey the underwater environment with great accuracy, especially in situations that defy classical technology. Namely, underwater surveys over rugged terrain and near vertical cliffs.

The WiMUST project (H2020-ICT-2014-1, 2015–2018) [15] witnessed the development of advanced cooperative and networked control/navigation systems to enable a group of marine robots (both on the surface and submerged) equipped with acoustic sources and towed acoustic streamers to perform geotechnical seismic surveys in a fully automatic manner. For the first time worldwide, a mission was performed in 2018 at sea in Sines, Portugal, with a fleet of seven autonomous marine robots performing high-resolution 3D sub-bottom mapping in cooperation. Every individual vehicle was required to be equipped with a path-following algorithm.

Path-following algorithms are fundamental for these vehicles to cooperate effectively. The straight-line path following problem for formations of multiple under-actuated marine surface vessels is addressed in [16]. The controller used is a combination of an LOS-based path-following controller and a nonlinear synchronization controller for the along-path synchronization of the vessels. The synchronization controller takes into account the loss of controllability at velocities close to zero for under-actuated vehicles. A unified analysis of stability properties of both the cross-track error dynamics and the synchronization error dynamics are discussed by using the tools from the theory of nonlinear cascaded systems. For the path-following controller of each vehicle, the authors used a line-of-sight guidance law in combination with a stabilizing heading controller. The guidance law is a function of cross-track error and look-ahead distance [17], which is an along-track distance between the nearest point on the track and a point that lies ahead of the vehicle. The look-ahead distance is used as design parameter. Depending on the damping on sway motion, the look-ahead distance can be increased or reduced to impose restrictions on the commanded yaw rate. However, the bound on the design parameter (look-ahead distance) requires the knowledge of the mass matrix and damping coefficients for surge and sway dynamics of the vehicle and the proposed controller does not take ocean currents into account.

Similar work that takes ocean currents into account is reported in [18]. The control law proposed to drive the cross-track error to zero is the same as that reported in [16] with an extra term which is a function of the ocean currents. The ocean currents for an individual vehicle are estimated using an adaptation law, which solves a differential Ricatti equation that is again a function of the mass matrix and damping coefficients.

In [19], instead of estimating the currents, the authors used an integrator similar to the work reported in this paper. Burger et al. introduced a conditional integrator to avoid large overshoots during the saturation of the control signal. The conditional integrator combines the benefits of integral action and sliding-mode control. It behaves either like a PI controller or like a sliding-mode controller, depending on the magnitude of the control signal to avoid the chattering caused by the sliding-mode control. However, the problem of integrator windup can also be addressed by using a smooth anti-windup scheme.

The tools used for stability analysis in [16,18,20] are similar to the one reported in this paper, which relies on the theory of interconnected and cascaded systems. In [20], path following problems for more general spatial paths (with constraints on their curvature) in the presence of constant ocean currents were addressed. The authors introduced a virtual Serret–Frenet reference frame that is anchored on and propagates along the desired path. When the vehicle reaches the vicinity of that point, the reference is updated, requesting the vehicle to converge to another point further on the reference path. A Luenberger-type observer is designed to estimate the currents by measuring the relative velocity of the vehicle w.r.t the water. The proposed guidance law is a nonlinear function of surge and sway velocities and involves solving a quadratic function of currents, cross-track error and look-ahead distance. The estimation of ocean currents requires the measurement of relative velocity from an Acoustic Doppler Current Profiler (ADCP). Most surface vehicles are equipped only with GPS and cannot measure the relative velocity of the vehicle. In the simplified case of [20], to follow straight lines while cruising at a constant speed, the measurement of the relative velocity was critical to estimate currents. Again, the design of the controller requires knowledge of surge and sway dynamics.

We now shift our attention to the importance of inner-outer loop control structures. In the field of aircraft control, path following has been addressed as dynamic and kinematic loop control structures similar to inner and outer loop in marine vehicles. In [21], path-following control in 3D was built on a nonlinear control strategy that is first derived at the kinematic level, followed by the design of a $L_{1}$-adaptive output-feedback control law that effectively augments an existing autopilot and yields an inner-outer loop control structure with guaranteed performance whereas, multiple vehicle coordination is achieved by enforcing temporal constraints on the speed profiles of the vehicles along their paths. A survey and analysis of algorithms for path following reported in [22] showed how inner-outer loop-based guidance schemes are implemented in most Unmanned Air Vehicles (UAV) where practitioners used inexpensive open-source autopilots.

There is extensive literature on the path following, displaying a vast choice of available control laws based on linear and nonlinear techniques. Representative examples of path-following controllers for marine vehicles can be found in [23,24,25,26]. Furthermore, in [27], the authors described a nonlinear path-following guidance method in inner-outer loop form, where the outer loop plays a role of a guidance scheme, generating lateral acceleration commands, and the inner loop follows. This paper does not address the stability of the outer loop in the presence of the inner loop. This topic was addressed in [28] with the assumption that there is complete knowledge of the vehicle model parameters.

No reference in the literature, to the best of our knowledge, addresses the problem of path following without prior knowledge of the inner loop dynamics. Path following is either designed at a kinematic level only or demands complete knowledge of horizontal plane dynamics.

The scarcity of publications on nonlinear path following for ocean vehicles without complete knowledge of vehicle dynamics somehow reflects the hardness of the problem mainly due to the presence of a nonzero lateral velocity and shows the relevance of the research topic here discussed. Motivated by the above considerations, this article addresses the problem of path following for marine vehicles by resorting to inner-outer control loops, with due account for the vehicle dynamics and currents. The inner-outer loop control structures exhibit a fast-slow temporal scale separation that yields simple “rules of thumb” for controller tuning. Stated intuitively, the inner loop dynamics should be much faster than those of the outer loop. This qualitative result is well rooted in singular perturbation theory [29]. Conceptually, the procedure described has three key advantages: (i) it decouples the design of the inner and outer control loops, (ii) the structure of the outer loop controller does not depend on the dynamics of the vehicle, and (iii) it provides practitioners a very convenient method to effectively implement path-following controllers on a wide range of vehicles.

The path-following controller discussed in this article is designed at the kinematic outer loop that commands the inner loop with the desired heading angles while the vehicle moves at an approximately constant speed. The idea is to provide a seamless implementation of path-following control algorithms on heterogeneous vehicles that may be pre-equipped with heading autopilots. To address this issue, we developed a novel methodology for the design of path-following controllers for marine vehicles which uses a simple characterization of the marine vehicle’s dynamics, in the form of input-output gains or bandwidth-like characterization, without having to know the detailed dynamics of a marine vehicle. This is the key contribution of this article, which is rooted in and extends substantially the methodology described in [30]. The focus of the presentation is on AUVs; however, the techniques can be easily extended to autonomous surface vehicles (ASVs). The paper is organized as follows. We first discuss the nonlinear dynamics of two marine vehicles used in experiments, followed by the formal proof of the stability of a simple inner-loop PD controller applied to a nonlinear three-degree-of-freedom model. We then tackle the problem of path following without considering the dynamics of the vehicle. In Section 6.5, we consider path following for straight lines in 2D, propose an inner-outer loop control structure for its solution, and provide the proof of the stability of the resulting feedback control system. We describe the results of simulations and field tests performed with real marine vehicles, summarize the main conclusions, and discuss problems that warrant further research.

## 2. Notation and AUV Modeling

Depth and heading controllers are the core systems of autonomous marine. Depth control is used to maintain the depth of an AUV at a desired value, whereas heading control is used to steer both AUVs and ASVs along desired directions with respect to the magnetic north. The design of such controllers varies from simple proportional-integral-derivative (PID) and linear quadratic methods based on linearized dynamic models [31] to more complex Lyapunov-based nonlinear control. Modeling the dynamics of a vehicle is critical for its maneuvering, stabilization, and motion control. However, accurate modeling of the dynamics of such vehicles is oftentimes painstaking, time consuming, and quite costly. In [32], the hydrodynamic data required to model the Marius AUV have been determined by full-scale tests, using a towing tank equipped with a *Planar Motion Mechanism*. There are only a few test facilities of this kind which many researchers developing AUVs for scientific needs cannot afford. To avoid such expensive and time-consuming methods of determining the hydrodynamic coefficients, most of the users rely on semi-empirical and analytical methods [33], together with CFD analysis. Later, the parameters of importance in simplified models can be derived/verified by performing certain open loop maneuvers in the water. One such example is the *circular maneuver* for horizontal plane models [34]. Vehicle models obtained using such techniques are necessarily simplified but, if properly exploited, may be extremely useful in characterizing the system to be controlled in a form that is suitable for input-output stability analysis. A compelling example is the case where, using nonlinear system analysis, the dynamics of a system may be characterized in terms of parameters that play a role equivalent to static gain and bandwidth for first-order linear systems. Such models can be used to design the controllers with a simple structure. In what follows, the structure of a generic vehicle model that we adapt borrows from the work of Fossen [34].

### 2.1. Vehicle Modeling

**Figure 2:**
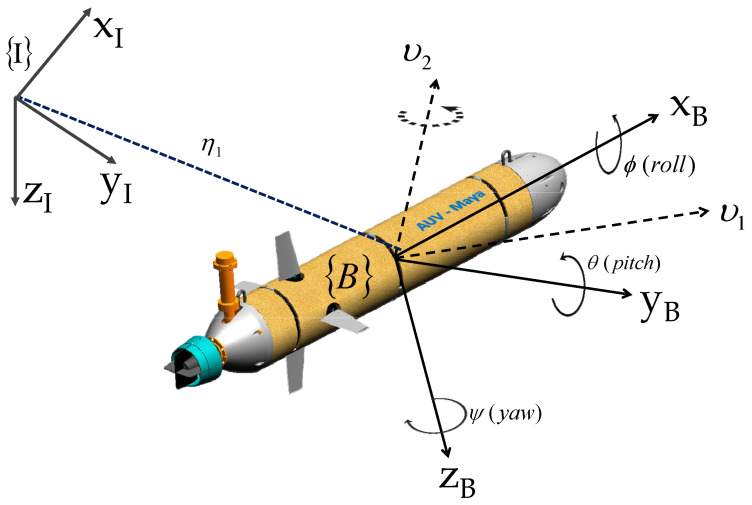
Notations and reference frames for an AUV.

Following usual practice, we define two reference frames: a body-fixed reference frame $\left\{B\right\}$ in which the dynamics of the vehicle are naturally described and an earth-fixed reference frame $\left\{I\right\}$ in which the position and orientation of the vehicle are expressed (see Figure 2). The following notation is required.

- $\nu_{1}={\left[u\;\;v\;\;w\right]}^{T}$ is the linear velocity of the origin of $\left\{B\right\}$ with respect to $\left\{I\right\}$ expressed in $\left\{B\right\}$ (i.e., body-fixed linear velocity).
- $\nu_{2}={\left[p\;\;q\;\;r\right]}^{T}$ is the angular velocity of $\left\{B\right\}$ with respect to $\left\{I\right\}$ expressed in $\left\{B\right\}$ (i.e., body-fixed angular velocity).
- $\eta_{1}={\left[x\;\;y\;\;z\right]}^{T}$ is the position of the origin of $\left\{B\right\}$ measured in $\left\{I\right\}$.
- $\eta_{2}={\left[\phi \;\;\theta \;\;\psi \right]}^{T}$ parameterizes locally the orientation of $\left\{B\right\}$ with respect to $\left\{I\right\}$.

An arbitrary vector ${}^{B}V\in {I\;R}^{3}$ expressed in $\left\{B\right\}$ can be expressed in $\left\{I\right\}$ as IV=BIRBV. An important relation for ${}_{B}^{I}R$ (abbreviated as *R*) [35] is $R^{T}R=I$, implying that $R^{T}=R^{-1}$ and det(*R*) =1. The matrix $R\in {I\;R}^{3\times 3}$ can be described locally in terms of a sequence of 3 transformations that take $\left\{B\right\}$ to $\left\{I\right\}$ by rotating it sequentially about its current z→y→x axis through the Euler angles ψ—yaw (rotation about *z*-axis), θ—pitch (rotation about *y*-axis) and ϕ—roll (rotation about *x*-axis) [34]. The final rotation matrix from $\left\{B\right\}$ to $\left\{I\right\}$ parameterized by the Euler angles $\eta_{2}={\left[\phi \;\;\theta \;\;\psi \right]}^{T}$ is given by

(1) $$\begin{array}{c} R\left(\eta_{2}\right)=\left(\begin{array}{ccc} c\psi c\theta & -s\psi c\phi +c\psi s\theta s\phi & s\psi s\phi +c\psi c\phi s\theta \\ s\psi c\theta & c\psi c\phi +s\phi s\theta s\psi & -c\psi s\phi +s\theta s\psi c\phi \\ -s\theta & c\theta s\phi & c\theta c\phi \end{array}\right), \end{array}$$

where s(.)=sin(.) and c(.)=cos(.).

### 2.2. Kinematics

The kinematic equations that relate body-fixed with inertial linear and angular velocities are given by

(2) $$\begin{array}{cc} {\dot{\eta }}_{1} & =R\left(\eta_{2}\right)\nu_{1}, \end{array}$$

(3) $$\begin{array}{cc} {\dot{\eta }}_{2} & =Q\left(\eta_{2}\right)\nu_{2}. \end{array}$$

The transformation of body-fixed angular velocities is performed using the transformation matrix $Q\left(\eta_{2}\right)$ given by

(4) $$\begin{array}{c} Q\left(\eta_{2}\right)=\left(\begin{array}{ccc} 1 & s\phi t\theta & c\phi t\theta \\ 0 & c\phi & -s\phi \\ 0 & s\phi /c\theta & c\phi /c\theta \end{array}\right), \end{array}$$

where t(.)=tan(.). Note that *Q* is singular for θ=±π/2 when using the above sequence of Euler angles and can be avoided by using quaternions (see [34] for details). However, for an AUV, an angle close to π/2 is practically not desirable and is avoided by design. Thus, it is reasonable to assume that most AUVs operate with small pitch angle. Finally, the combined 6-DOF kinematic equation can be written as

(5) $$\begin{array}{cc} \left[\begin{array}{c} \dot{\eta_{1}}\\ \dot{\eta_{2}} \end{array}\right] & =\left[\begin{array}{cc} R\left(\eta_{2}\right) & 0\\ 0 & Q\left(\eta_{2}\right) \end{array}\right]\left[\begin{array}{c} \nu_{1}\\ \nu_{2} \end{array}\right]⟺\dot{\eta }=J\left(\eta \right)\nu . \end{array}$$

An important relation for the derivative of a rotation matrix is given by

(6) $$\begin{array}{cc} \dot{R}\left(\eta_{2}\right) & =R\left(\eta_{2}\right)S\left(\nu_{2}\right), \end{array}$$

where *S* is a skew-symmetric matrix, for an arbitrary vector $u={\left[u_{x}\;u_{y}\;u_{z}\right]}^{T}\;\;\in {I\;R}^{3}$, it takes the form

(7) $$\begin{array}{c} S\left(u\right)=\left[\begin{array}{ccc} 0 & -u_{z} & u_{y}\\ u_{z} & 0 & -u_{x}\\ -u_{y} & u_{x} & 0 \end{array}\right]. \end{array}$$

Furthermore,

(8) $$\begin{array}{c} S^{T}=-S;\;\;S\left(u\right)v=-S\left(v\right)u;\;\;S\left(u\right)v=u\times v. \end{array}$$

### 2.3. Dynamics

With the assumptions that the center of mass of the rigid body is coincident with the origin of $\left\{B\right\}$ and $\left\{I\right\}$ is an inertial frame, Newton–Euler’s laws apply in the latter frame and the dynamic equations for translation can be written as

(9) $$\begin{array}{cc} \sum {}^{I}F_{RB} & =m\frac{d}{dt}\left({}^{I}\nu_{1}\right)=m\frac{d}{dt}\left(R\nu_{1}\right) \end{array}$$

(10) $$\begin{array}{cc} \; & =m\frac{dR}{dt}\nu_{1}+mR\frac{d}{dt}\nu_{1} \end{array}$$

(11) $$\begin{array}{cc} \; & =mRS\left(\nu_{2}\right)\nu_{1}+mR\frac{d}{dt}\nu_{1} \end{array}$$

where *m* is the mass matrix and $\sum^{I}F_{RB}$ is the sum of external forces expressed in the inertial reference frame. The dynamic equations for an AUV are usually expressed in the body-fixed frame for convenience, where the inertia tensor is constant and the external forces are more easily expressed. The sum of external forces expressed in $\left\{B\right\}$ is given by

(12) $$\begin{array}{cc} \sum R^{-1}{}^{I}F_{RB} & =mR^{-1}RS\left(\nu_{2}\right)\nu_{1}+mR^{-1}R\frac{d}{dt}\nu_{1} \end{array}$$

(13) $$\begin{array}{cc} \sum F_{RB} & =m\left[\nu_{2}\times \nu_{1}+{\dot{\nu }}_{1}\right], \end{array}$$

where $F_{RB}$ are the external forces measured in $\left\{B\right\}$. Similarly, applying Newton–Euler’s laws in $\left\{B\right\}$, the dynamic equations for rotational motion can be written as

(14) $$\begin{array}{cc} \sum {}^{I}N_{RB} & =\frac{d}{dt}\left({}^{I}L\right)=\frac{d}{dt}\left(RI_{RB}\nu_{2}\right) \end{array}$$

(15) $$\begin{array}{cc} & =RS\left(\nu_{2}\right)I_{RB}\nu_{2}+RI_{RB}\frac{d}{dt}\nu_{2}, \end{array}$$

where $I_{RB}$ is the moment of inertia matrix and ${}^{I}L$ is the angular momentum measured in $\left\{I\right\}$. Now, expressing the above dynamic equations in body-fixed reference frame $\left\{B\right\}$ yields

(16) $$\begin{array}{cc} \sum R^{-1}{}^{I}N_{RB} & =R^{-1}RS\left(\nu_{2}\right)I_{RB}\nu_{2}+R^{-1}RI_{RB}\frac{d}{dt}\nu_{2} \end{array}$$

(17) $$\begin{array}{cc} \sum N_{RB} & =I_{RB}{\dot{\nu }}_{2}+\nu_{2}\times I_{RB}\nu_{2}, \end{array}$$

where $N_{RB}$ includes the external torques measured in $\left\{B\right\}$.

Combining the equations for translation and rotational motion, a simplified vectorial representation can be written as

(18) $$\begin{array}{cc} M_{RB}\dot{\nu }+C_{RB}\left(\nu \right)\nu & =\tau_{RB}, \end{array}$$

where $M_{RB}$ is the rigid-body inertia matrix, which in the general case, is given by

(19) $$\begin{array}{cc} M_{RB} & =\left[\begin{array}{cc} mI_{3\times 3} & -mS\left(r_{g}^{b}\right)\\ mS\left(r_{g}^{b}\right) & I_{o} \end{array}\right] \end{array}$$

(20) $$\begin{array}{cc} & =\left[\begin{array}{cccccc} m & 0 & 0 & 0 & mz_{g} & -my_{g}\\ 0 & m & 0 & -mz_{g} & 0 & -mx_{g}\\ 0 & 0 & m & my_{g} & -mx_{g} & 0\\ 0 & -mz_{g} & my_{g} & I_{x} & -I_{xy} & -I_{xz}\\ mz_{g} & 0 & -mx_{g} & -I_{xy} & I_{y} & -I_{yz}\\ -my_{g} & mx_{g} & 0 & -I_{zx} & -I_{zy} & I_{z} \end{array}\right], \end{array}$$

and satisfies the properties

- $M_{RB}=M_{RB}^{T}>0$,
- ${\dot{M}}_{RB}=0_{6\times 6}$,

where $I_{3\times 3}$ is the identity matrix, $I_{o}=I_{o}^{T}>0$ is the inertia matrix about O, and $S\left(r_{g}^{b}\right)$ in (19) is the matrix cross-product operator.

The rigid body Coriolis and centripetal matrix $C_{RB}\left(\nu \right)$ can always be represented in symmetric form, i.e., $C_{RB}\left(\nu \right)=C_{RB}^{T}\left(\nu \right)\;\forall \nu \in {I\;R}^{6}$. For the given inertia matrix,

(21) $$\begin{array}{c} M_{RB}=M_{RB}^{T}=\left[\begin{array}{cc} M_{11} & M_{12}\\ M_{21} & M_{22} \end{array}\right]>0, \end{array}$$

where $M_{21}=M_{12}^{T}$, $C_{RB}\left(\nu \right)$ can be written as

(22) $$\begin{array}{c} C_{RB}\left(\nu \right)=\left[\begin{array}{cc} 0_{3\times 3} & -mS\left(\nu_{1}\right)-mS\left(S\left(\nu_{2}\right)r_{g}^{b}\right)\\ -mS\left(\nu_{1}\right)-mS\left(S\left(\nu_{2}\right)r_{g}^{b}\right) & mS\left(S\left(\nu_{1}\right)r_{g}^{b}\right)-S\left(I_{o}\nu_{2}\right) \end{array}\right] \end{array}$$

with $S\left(\nu_{1}\right)\nu_{1}=0$. All external forces and torques are represented as a generalized vector $\tau_{RB}={\left[\sum F_{RB}^{T}\;\;\sum N^{T}RB\right]}^{T}={\left[X\;Y\;Z\;K\;M\;N\right]}^{T}$ where ${\left[X\;Y\;Z\right]}^{T}$ are the external forces and ${\left[K\;M\;N\right]}^{T}$ are the external torques both expressed in body $\left\{B\right\}$. To explicitly take into account different types of external forces and torques, this vector can be decomposed as

(23) $$\begin{array}{cc} \tau_{RB} & =\tau +\tau_{A}+\tau_{D}+\tau_{R}+\tau_{dist}, \end{array}$$

where

- τ—control inputs (forces and torques due to thrusters/surfaces);
- $\tau_{A}=-M_{A}\dot{\nu }-C_{A}\left(\nu \right)\nu$—terms due to added masses;
- $\tau_{D}=-D\left(\nu \right)\nu$—hydrodynamics terms due to lift, drag, skin friction, etc.;
- $\tau_{R}=-g\left(\eta \right)$—restoring forces and torques due to the interplay between gravity and buoyancy forces;
- $\tau_{dist}$—terms due to external disturbances, e.g., waves, winds, etc.

Neglecting the term $\tau_{dist}$, the final dynamic model of an AUV can be written as

(24) $$\begin{array}{cc} \left[M_{RB}+M_{A}\right]\dot{\nu }+\left[C_{RB}\left(\nu \right)+C_{A}\left(\nu \right)\right]\nu +D\left(\nu \right)\nu +g\left(\eta \right) & =\tau \end{array}$$

(25) $$\begin{array}{cc} \Leftrightarrow M\dot{\nu }+C\left(\nu \right)\nu +D\left(\nu \right)\nu +g\left(\eta \right) & =\tau . \end{array}$$

## 3. Examples of Horizontal Plane Dynamics

### 3.1. 3-DOF Nonlinear Model

In what follows, we consider the horizontal plane dynamics of an underwater vehicle with 3 degrees of freedom (surge, sway, and yaw rate). Assuming that the roll of the vehicle is negligible and vertical and the horizontal plane dynamics are decoupled, and the $\left\{B\right\}$ frame coincides with the principal axes of inertia of the body, the corresponding nonlinear equations of motion can be written as

(26) $$\begin{array}{cc} m_{u}\dot{u}-m_{v}vr+d_{u}u & =\tau_{u} \end{array}$$

(27) $$\begin{array}{cc} m_{v}\dot{v}+m_{u}ur+d_{v}v & =0 \end{array}$$

(28) $$\begin{array}{cc} m_{r}\dot{r}-m_{uv}uv+d_{r}r & =\tau_{r} \end{array}$$

with

(29) $$\begin{array}{cccccc} m_{u} & =m-X_{\dot{u}} & m_{r} & =I_{z}-N_{\dot{r}} & d_{u} & =-X_{u}-X_{|u|u}\left|u\right| \end{array}$$

(30) $$\begin{array}{cccccc} m_{v} & =m-Y_{\dot{v}} & m_{uv} & =m_{u}-m_{v} & d_{v} & =-Y_{v}-Y_{|v|v}\left|v\right| \end{array}$$

(31) $$\begin{array}{cccccc} \; & \; & & \; & d_{r} & =-N_{r}-N_{|r|v}\left|r\right|, \end{array}$$

where *m* is the mass, $I_{zz}$ is the moment of inertia about the vertical axis, $X_{\dot{u}}$, $Y_{\dot{v}}$, and $N_{\dot{r}}$ are added mass coefficients, $X_{u}$, $Y_{v}$, and $N_{r}$ are linear damping coefficients, and $X_{|u|u}$, $Y_{|v|v}$, and $N_{|r|r}$ are the nonlinear damping coefficients of the AUV model. The compact form of these equations describing the motion of a marine vehicle which is three-plane symmetric can be written as [36]

(32) $$\begin{array}{c} M\dot{\nu }+C\left(\nu \right)\nu +D\left(\nu \right)\nu =\tau , \end{array}$$

where

(33) $$\begin{array}{cccc} \nu & ={\left[u\;\;\;v\;\;\;r\right]}^{T}, & \tau & ={\left[\tau_{u}\;\;\;0\;\;\tau_{r}\right]}^{T}, \end{array}$$

(34) $$\begin{array}{cc} M & =\left(\begin{array}{ccc} m_{u} & 0 & 0\\ 0 & m_{v} & 0\\ 0 & 0 & m_{r} \end{array}\right)>0, \end{array}$$

(35) $$\begin{array}{cc} C\left(\nu \right) & =\left(\begin{array}{ccc} 0 & 0 & -m_{v}v\\ 0 & 0 & m_{u}u\\ m_{v}v & -m_{u}u & 0 \end{array}\right), \end{array}$$

(36) $$\begin{array}{cc} D\left(\nu \right) & =\left(\begin{array}{ccc} d_{u} & 0 & 0\\ 0 & d_{v} & 0\\ 0 & 0 & d_{r} \end{array}\right)>0, \end{array}$$

**Figure 3:**
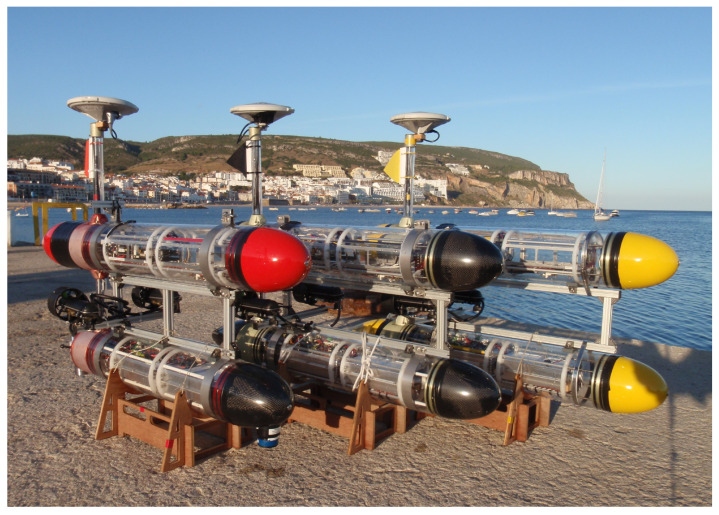
Medusa Autonomous Marine Vehicles, developed at DSOR, IST, Lisbon.

**Figure 4:**
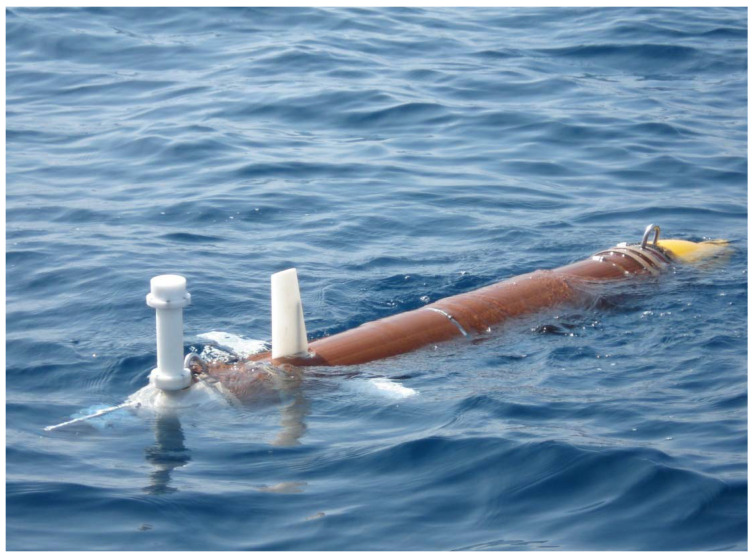
The MAYA Autonomous underwater vehicle developed at NIO, Goa.

We shall now discuss the modeling of an underwater vehicle referring to two examples of actual vehicles: (1) the MEDUSA vehicle in which yaw and heave motions are controlled only by thrusters (see Figure 3), (2) Maya, a torpedo-shaped AUV where the yaw and pitch motions are controlled using conventional rudders and fins while the vehicle cruises propelled by a single thruster aligned with the *x*-axis of the body (see Figure 4). In this model, the surge equations are not considered, and the vehicle is assumed to be cruising at a constant speed $u_{o}$ [31].

### 3.2. MEDUSA-Class Vehicle as an Example

The MEDUSA-class vehicles are autonomous robotic marine vehicles, capable of working both as surface and underwater robots, developed at the Dynamical Systems and Ocean Robotics group (DSOR) of Instituto de Sistemas e Robótica, Instituto Superior Técnico (ISR-IST) [37]. The MEDUSA-class AUV is a twin hull vehicle separated by 150 mm (see Figure 3). It weighs around 17 kg, is 1 m long, and with a hull diameter of 150 mm. The two hulls contain the batteries, onboard electronics, sensors, and the main computer running the Robot Operating System (ROS).

The surface operating vehicles have two thrusters placed on each side of the vehicle at 150 mm from the center line. The forward force $\tau_{u}=F_{s}+F_{p}$ is generated as a sum of two forces generated by starboard ($F_{s}$ ) and portside ($F_{p}$) thrusters and a yaw moment $\tau_{r}=0.15\left(F_{s}-F_{p}\right)$. The restoring moments for the vehicle is large enough for the roll and pitch motions to be neglected due to large separation between center of gravity and center of buoyancy. The vehicles are not actuated in the sway axis (i.e., $\tau_{v}=0$) [38]. The hydrodynamic parameters for Medusa are derived using the combination of semi-empirical and analytical methods and experimental data in calm waters. The parameters are tabulated in Table A1.

### 3.3. Maya AUV: An Example

The Maya AUV is an axis-symmetric underwater vehicle developed at the National Institute of Oceanography (NIO), Goa, India. The Maya AUV [39] follows a low-drag hull with a removable nose cone which carries scientific sensors (see Figure 2 and Figure 4). It has a single propeller for propulsion and two pairs of stern planes to control depth and heading. The nose section can accommodate different sensors for specific missions at sea. The AUV is equipped with an attitude and heading reference system (AHRS), a Doppler velocity log (DVL) for navigation underwater, and GPS for surface navigation.

Based on the assumption that the complete six-degrees-of-freedom model for the AUV can be split into two non-interacting models for the vertical and horizontal planes (see [40]), the simplified sway and yaw dynamics at constant speed $u_{0}$ are given by [36],

(37) $$\begin{array}{cc} m\dot{v}+mu_{0}r & =Y \end{array}$$

(38) $$\begin{array}{cc} I_{z}\dot{r} & =N. \end{array}$$

For small roll and pitch angles,

(39) $$\begin{array}{c} \dot{\psi }=\frac{sin\theta }{cos\theta }q+\frac{cos\phi }{cos\theta }r\approx r. \end{array}$$

The linear modeling of hydrodynamic damping, added mass, and rudder angle gives

(40) $$\begin{array}{cc} Y & =Y_{v}\dot{v}+Y_{R}\dot{r}+Y_{v}v+Y_{r}r+Y_{\delta }\delta_{r} \end{array}$$

(41) $$\begin{array}{cc} N & =N_{v}\dot{v}+N_{R}\dot{r}+N_{v}v+N_{r}r+N_{\delta }\delta_{r}. \end{array}$$

The quadratic terms on damping are neglected because of limited magnitude of *v* and *r*. The model is linearized about the nominal cruising speed of $u_{0}=1.2$ m/s and the linearized dynamic equations of motion for the horizontal plane represented in matrix form is given by

(42) $$\begin{array}{c} \left[\begin{array}{ccc} m-Y_{\dot{v}} & -Y_{\dot{r}} & 0\\ -N_{\dot{v}} & I_{z}-N_{\dot{r}} & 0\\ 0 & -1 & 1 \end{array}\right]\left[\begin{array}{c} \dot{v}\\ \dot{r}\\ \dot{\psi } \end{array}\right]+\left[\begin{array}{ccc} -Y_{v} & -Y_{r}+mu_{0} & 0\\ -N_{v} & -N_{r} & 0\\ 0 & -1 & 0 \end{array}\right]\left[\begin{array}{c} v\\ r\\ \psi \end{array}\right]=\left[\begin{array}{c} Y_{\delta }\\ N_{\delta }\\ 0 \end{array}\right]\delta_{r}. \end{array}$$

The vehicle parameters were estimated by resorting to analytic and semi-empirical methods for hydrodynamic parameter estimation (see [33]), and the details of the parameters for the MAYA AUV are given in Table A2.

## 4. Heading Control for a 3-DOF Nonlinear Model

In preparation for the analysis of combined guidance and control systems for a marine vehicle in 2D, we start by obtaining a compact description of the dynamics of a closed-loop yaw control system. This will serve as an important step to tackle the problem of path following. Most surface and underwater vehicles use simple Proportional-Derivative (PD) yaw controllers, oftentimes designed using a 3-DOF model linearized about a trimming condition with a view to steering the vehicle along a straight line at a fixed forward speed. However, there is no formal proof of stability for the convergence of the error (between desired and true heading) to zero when the controller is applied to a nonlinear model. This section provides proof of convergence for a PD controller coupled with the 3-DOF nonlinear dynamics of a Medusa Class marine vehicle, using concepts from the Lyapunov theory. We will be later using a simple characterization of the combination of the AUV dynamics with a heading controller, in the form input-output gains or bandwidth-like characterization and this is a key contribution of this article. The motion of an AUV in the horizontal plane expressed in the body-fixed frame is given in (27), and is repeated here for the reader’s convenience:

(43) $$M\dot{\nu }+C\left(\nu \right)\nu +D\left(\nu \right)\nu =\tau ,$$

where $\nu ={\left[u\;v\;r\right]}^{T}$ is the state vector; $\tau ={\left[\tau_{u}\;0\;\tau_{r}\right]}^{T}$ is the control vector and

$$M=\left[\begin{array}{ccc} m_{u} & 0 & 0\\ 0 & m_{v} & 0\\ 0 & 0 & m_{r} \end{array}\right]>0,$$

$$C\left(\nu \right)=\left[\begin{array}{ccc} 0 & 0 & -m_{v}v\\ 0 & 0 & m_{u}u\\ m_{v}v & -m_{u}u & 0 \end{array}\right],$$

$$D\left(\nu \right)=\left[\begin{array}{ccc} d_{u} & 0 & 0\\ 0 & d_{v} & 0\\ 0 & 0 & d_{r} \end{array}\right]>0,$$

$m_{u}=m-X_{\dot{u}}$; $m_{v}=m-Y_{\dot{v}}$; $m_{r}=I_{zz}-N_{\dot{r}}$,

$d_{u}=-X_{u}-X_{|u|u}\left|u\right|=d_{u1}+d_{u2}\left|u\right|$,

$d_{v}=-Y_{v}-Y_{|v|v}\left|v\right|=d_{v1}+d_{v2}\left|v\right|$,

$d_{r}=-N_{r}-N_{|r|r}\left|r\right|=d_{r1}+d_{r2}\left|r\right|$.

Considering practical issues, the following assumptions are made:

(a) The surge velocity *u* is much larger than the sway *v* so that u≈U is constant, where *U* is the total speed of the vehicle w.r.t the fluid;
(b) The yaw rate and therefore yaw are controlled using a proportional-derivative control law given by, i.e., $\tau_{r}=-K\tilde{\psi }-K_{d}\left(r-{\dot{\psi }}_{d}\right)$, where $\tilde{\psi }=\psi -\psi_{d}$ is the negative of the yaw heading error, and $\psi_{d}$ is the heading command;
(c) The dynamics of the heading is replaced by the dynamics of the heading error, i.e., $\dot{\tilde{\psi }}=\dot{\psi }-\dot{\psi_{d}}$.

Thus, taking into account assumptions (a) to (c), the mathematical model of the system that describes, for a fixed *U*, the evolution of the closed-loop system under PD control, is given by

(44) $$\begin{array}{cc} \left[\begin{array}{c} \dot{v}\\ \dot{r}\\ \dot{\tilde{\psi }} \end{array}\right]= & \underset{A}{\underset{︸}{\left[\begin{array}{ccc} -\frac{d_{v1}}{m_{v}} & -\frac{m_{u}}{m_{v}}U & 0\\ -\frac{m_{v}-m_{u}}{m_{r}}U & -\frac{d_{r1}+K_{d}}{m_{r}} & -\frac{K}{m_{r}}\\ 0 & 1 & 0 \end{array}\right]}}\underset{x}{\underset{︸}{\left[\begin{array}{c} v\\ r\\ \tilde{\psi } \end{array}\right]}}+\\ \; & \underset{G(x)}{\underset{︸}{\left[\begin{array}{ccc} -\frac{d_{v2}\left|v\right|}{m_{v}} & 0 & 0\\ 0 & -\frac{d_{r2}\left|r\right|}{m_{r}} & 0\\ 0 & 0 & 0 \end{array}\right]}}\left[\begin{array}{c} v\\ r\\ \tilde{\psi } \end{array}\right]+\underset{B}{\underset{︸}{\left[\begin{array}{c} 0\\ \frac{K_{d}}{m_{r}}\\ -1 \end{array}\right]}}{\dot{\psi }}_{d} \end{array}$$

Equation (44) can be rewritten as:

(45) $$\dot{x}=Ax+G\left(x\right)x+Bu,\;u\in I\;R$$

for $x\in D_{x}=\{v,r,\tilde{\psi }\subset {I\;R}^{3}:|v|\leq v_{max}\;|r|\leq r_{max},\;|\tilde{\psi }\left|\leq {\tilde{\psi }}_{max}\right\}$ and $u\in D_{u}=\{u\subset I\;R:|u|\leq u_{max}\}$. Notice that Equation (45) contains linear and nonlinear terms.

## 5. Analysis of the Stability of the Origin

The stability analysis of the origin of the mathematical model of the system (45) is performed considering the influence of the vehicle speed *U* on the eigenvalues of matrix *A*, once the values for *K* and $K_{d}$ have been defined. To analyze the stability of the origin, we use the properties of *input-to-state* (ISS) and *input-output stability* (IOS) of the control system. Furthermore, this analysis is important because ISS and IOS properties of the system are based on a Lyapunov equation that involves the matrix *A*. In what follows, we provide definitions of ISS and IOS and essential theorems from [29] that will be used to perform the stability analysis of the systems considered in this paper.

### 5.1. Input-to-State Stability (ISS)

Consider the system

(46) $$\dot{x}=f\left(x,t,u\right)\;x\left(t_{0}\right)=x_{0}$$

where $x\left(t\right)\in {I\;R}^{n}$ and $u\left(t\right)\in {I\;R}^{m}$ denotes states and the input at time t≥0. The function $f:\left[0,\infty \right]\times {I\;R}^{n}\times {I\;R}^{m}\to {I\;R}^{n}$ is piecewise continuous in *t* and locally Lipschitz in *x* and *u*. The input u(t) is a piecewise continuous, bounded function of *t* for all t≥0.

**Definition** **1.** *The system* (46) *is said to be input-to-state stable (ISS) if there exists a class KL-function β and α class K-function, such that for any initial slate $x\left(t_{0}\right)$ and any bounded input $u\left(t\right)$, the solution $x\left(t\right)$ exists for all $t\geq t_{0}$ and satisfies*

(47) $$∥x\left(t\right)∥\leq \beta \left(∥x_{0}∥,t-t_{0}\right)+\gamma \left(\underset{t_{0}\leq \tau \leq t}{\sup}∥u\left(\tau \right)∥\right)$$

*The function γ∈K describes the influence of the input on the solution of the system. The function β∈KL describes the transient behavior of the system* [29,41].

The Lyapunov-based technique for the ISS verification gives a sufficient condition for input-to-state stability.

**Theorem** **1.** *Let* $V:\left[0,\infty \right)\times {I\;R}^{n}\to I\;R$ *be a continuously differentiable function such that*

(48) $$\alpha_{1}\left(∥x∥\right)\leq V\left(t,x\right)\leq \alpha_{2}\left(∥x∥\right)$$

(49) $$\frac{\partial v}{\partial t}+\frac{\partial v}{\partial x}f\left(x,t,u\right)\leq -W_{3}\left(x\right),\;\;\forall \;\;∥x∥\geq \rho \left(∥u∥\right)>0$$

$\forall \left(t,x,u\right)\epsilon \left[0,\infty \right)\times {I\;R}^{m}\times {I\;R}^{n}$ *, where* $\alpha_{1},\alpha_{2}$ *are class* $K_{\infty }$ *-functions, ρ is class* K *-function and* $W_{3}\left(x\right)$ *is continuous definite function on* ${I\;R}^{n}$ *. Then, the system *(46)* is input-to-state stable with* $\gamma =\alpha_{1}^{-1}\;o\;\alpha_{2}\;o\;\rho$ *.*

### 5.2. Input-Output Stability (IOS)

To understand the notion of input-output stability, we start by considering a system *H* with input *u* and output *y*. The system *H* is a map between two signals spaces y=H(u). The gain γ of *H* measures the largest amplification from *u* to *y*, where the magnitude of the latter is computed using appropriate function norms, see [29]. *H* can be a constant, a matrix, a linear system or a nonlinear system. The gain γ of *H* is defined as

(50) $$\gamma \left(H\right)=\underset{u\in L_{p}}{\sup}\frac{{∥y∥}_{p}}{{∥u∥}_{p}}=\underset{u\in L_{p}}{\sup}\frac{{∥H(u)∥}_{p}}{{∥u∥}_{p}}.$$

The system *H* is finite-gain *bounded-input bounded-output* (BIBO) if γ(H)<∞.

The above stability concept has been extended to capture the effect of initial conditions and possible biases in the input-output operator, yielding the concept of input-to-output (IOS) stability. The resulting concept and main stability result, taken from [29], are briefly summarized next. In this context, Lyapunov stability tools can be used to establish the IOS stability of nonlinear systems represented by state models. Consider a state model presented in (46), together with output function

(51) $$y=h\left(x,t,u\right)$$

where $h:\left[0,\infty \right]\times D\times D_{u}\to {I\;R}^{q}$ is piecewise continuous in *t* and continuous in (x,u), where $D\subset {I\;R}^{n}$ is a domain that contains x=0, and $D_{u}\subset {I\;R}^{m}$ is a domain that contains u=0. The following theorem states conditions under which, following the terminology in Khalil, a system is L-stable or a small signal is L-stable for a given choice of signal space L. Suppose x=0 is an equilibrium point of the unforced system

(52) $$\dot{x}=f\left(t,x,0\right).$$

**Theorem** **2.** *Consider the system *(46)* and *(51)* and take* r>0 *and* $r_{u}>0$ *such that* $\left\{∥x∥\leq r\right\}\subset D$ *and* $\left\{∥u∥\leq r_{u}\right\}\subset D_{u}$ *. Suppose that*

-
x=0 *is an equilibrium point of *(52)*, and there is Lyapunov function* $V\left(t,x\right)$ *that satisfies*
(53) $$c_{1}{∥x∥}^{2}\leq V\left(t,x\right)\leq c_{2}{∥x∥}^{2}$$
(54) $$\frac{\partial v}{\partial t}+\frac{\partial v}{\partial x}f\left(t,x,0\right)\leq -c_{3}{∥x∥}^{2}$$
(55) $$∥\frac{\partial v}{\partial x}∥\leq c_{4}∥x∥$$
  *for all* $\left(t,x\right)\in \left[0,\infty \right)\times D$ *for some positive constants* $c_{1},\;c_{2},\;c_{3},$ *and* $c_{4}$ *.*
- *f and h satisfy the inequalities*
(56) $$∥f\left(t,x,u\right)\;-\;f\left(t,x,0\right)∥\leq L∥u∥$$
(57) $$∥h\left(t,x,u\right)∥\leq \eta_{1}∥x∥+\eta_{2}∥u∥$$
  *for all* $\left(t,x\right)\in \left[0,\infty \right)\times D\times D_{u}$ *for some non negative constants* $L,\;\eta_{1},$ *and* $\eta_{2}$ *.*

*Then, for each* $x_{0}$ *with* $∥x∥\leq r\sqrt{c_{1}/c_{2}}$ *, the system *(46)* and *(51)* is small-signal finite gain* $L_{p}$ *-stable for each* $p\in \left[1,\infty \right]$ *. In particular, for each* $u\in L_{pe}$ *with* $\sup_{t_{0}\leq \tau \leq t}∥u\left(t\right)∥\leq min\left\{r_{u},c_{1}c_{3}r/\left(c_{2}c_{4}L\right)\right\}$ *, the output* y(t) *satisfies*

(58) $$∥y_{\tau }∥\leq \gamma {∥u_{\tau }∥}_{L_{p}}+\beta$$

*for all* $\tau \in \left[0,\infty \right)$ *, with*

(59) $$\gamma =\eta_{2}+\frac{\eta_{1}c_{2}c_{4}L}{c_{1}c_{3}},$$

(60) $$\beta =\eta_{1}∥x_{0}∥\sqrt{\frac{c_{2}}{c_{1}}}\rho ,\;where\;\rho =\{\begin{array}{cc} 1 & if\;p=\infty \\ {\left(\frac{2c_{2}}{c_{3}p}\right)}^{\left(1/p\right)}, & if\;p\in [1,\infty ) \end{array}$$

*Furthermore, if the origin is globally exponentially stable and the assumptions hold globally (with* $D={I\;R}^{n}$ *and* $D_{u}={I\;R}^{m}$ *), then, for each* $x_{0}\in {I\;R}^{n}$ *, the system *(46)* and *(51)* is finite gain* $L_{p}$ *-stable for each* $p\in \left[1,\infty \right]$ *.*

We refer the reader to explore [29] for details of the proof. ISS and IOS are the key Lyapunov stability tools used further for the analysis of our system.

### 5.3. ISS Analysis

For ISS analysis, the system model should satisfy the following equations for a suitably defined Lyapunov function candidate V(t,x):

(61) $$\alpha_{1}\left(∥x∥\right)\leq V\left(t,x\right)\leq \alpha_{2}\left(∥x∥\right)$$

(62) $$\frac{\partial V}{\partial t}+\frac{\partial V}{\partial x}f\left(t,x,u\right)\leq -W_{3}\left(x\right)\;\forall ∥x∥\geq \rho \left(∥x∥\right)>0.$$

where in this particular case, f(t,x,u) is the right-hand side of (45). Then, the system is ISS with, $\gamma =\alpha_{1}^{-1}\circ \alpha_{2}\circ \rho$ (please refer to [29] for definitions and theorems mentioned from now on).

Consider the following Lyapunov function candidate to check the ISS property of the system, given by

(63) $$V=x^{T}Px$$

where *P* is a nonsingular symmetric matrix.

Then,

(64) $$\lambda_{min}{\left(P\right)∥x∥}^{2}\leq x^{T}Px\leq \lambda_{max}\left(P\right){∥x∥}^{2}$$

Thus, comparing (61) with Equation (64), we conclude that

(65) $$\alpha_{1}\left(∥x∥\right)=\lambda_{min}\left(P\right){∥x∥}^{2}$$

(66) $$\alpha_{2}\left(∥x∥\right)=\lambda_{max}\left(P\right){∥x∥}^{2}$$

The derivative of V along the trajectories defined by Equation (45) is given by

(67) $$\dot{V}=x^{T}\left[{A+G\left(x\right)]}^{T}P+P[A+G\left(x\right)\right]x+2uB^{T}Px$$

Equation (67) can be rewritten as

(68) $$\dot{V}=x^{T}\left(A^{T}P+PA\right)x+2x^{T}PG\left(x\right)x+2uB^{T}Px.$$

The matrix product PG(x) results in

(69) $$F=PG=\left[\begin{array}{ccc} -p_{1,1}d_{1}\left|x_{1}\right| & -p_{1,2}d_{2}\left|x_{2}\right| & 0\\ -p_{1,2}d_{1}\left|x_{1}\right| & -p_{2,2}d_{2}\left|x_{2}\right| & 0\\ -p_{1,3}d_{1}\left|x_{1}\right| & -p_{2,3}d_{2}\left|x_{2}\right| & 0 \end{array}\right]=\left[\begin{array}{ccc} {\bar{f}}_{1,1}\left|x_{1}\right| & {\bar{f}}_{1,2}\left|x_{2}\right| & 0\\ {\bar{f}}_{2,1}\left|x_{1}\right| & {\bar{f}}_{2,2}\left|x_{2}\right| & 0\\ {\bar{f}}_{3,1}\left|x_{1}\right| & {\bar{f}}_{3,2}\left|x_{2}\right| & 0 \end{array}\right]$$

where $d_{1}=\frac{d_{v2}}{m_{v}}$ and $d_{2}=\frac{d_{r2}}{m_{r}}$, and $p_{ij}$ is the entry i,j of *P*. Inserting both the Lyapunov equation $A^{T}P+PA=-Q$ and $x^{T}Fx$ into Equation (68) yields

(70) $$\begin{array}{cc} \dot{V}= & -x^{T}Qx+2[{\bar{f}}_{1,1}|x_{1}|x_{1}^{2}+{\bar{f}}_{2,2}|x_{2}|x_{2}^{2}+({\bar{f}}_{2,1}|x_{1}|+{\bar{f}}_{1,2}|x_{2}\left|\right)x_{1}x_{2}+{\bar{f}}_{3,1}\left|x_{1}\right|x_{3}x_{1}\\ & +{\bar{f}}_{3,2}\left|x_{2}\right|x_{3}x_{2}]+2uB^{T}Px \end{array}$$

The following inequalities can be used in Equation (70):

(I) ${\bar{f}}_{1,1}\left|x_{1}\right|x_{1}^{2}\leq 0$ and ${\bar{f}}_{2,2}\left|x_{2}\right|x_{2}^{2}\leq 0$ because $p_{1,1}\geq 0$ and $p_{2,2}\geq 0$;
(II) $x_{1}x_{2}+x_{1}x_{3}+x_{2}x_{3}\leq x_{1}^{2}+x_{2}^{2}+x_{3}^{2}={∥x∥}^{2}$.
  This follows from the inequality ${\left(x_{1}-x_{2}\right)}^{2}+{\left(x_{1}-x_{3}\right)}^{2}+{\left(x_{2}-x_{3}\right)}^{2}\geq 0$.

Taking into account inequality I in Equation (70) yields

(71) $$\begin{array}{cc} \dot{V}\leq & -x^{T}Qx+2\left[|({\bar{f}}_{2,1}|x_{1}|+{\bar{f}}_{1,2}|x_{2}\left|)|\right|x_{1}x_{2}\left|+\right|{\bar{f}}_{3,1}\left|\right|x_{1}\left|\right|x_{3}x_{1}\left|+\right|{\bar{f}}_{3,2}\left|\right|x_{2}\left|\right|x_{3}x_{2}|\right]\\ & +2∥u∥∥B^{T}∥∥P∥∥x∥ \end{array}$$

Now, inserting inequality II into Equation (71) leads to

(72) $$\dot{V}\leq -\lambda_{min}{\left(Q\right)∥x∥}^{2}+2\left[\left(\right|{\bar{f}}_{2,1}\left|+\right|{\bar{f}}_{3,1}\left|)\right|x_{1}\left|+(\right|{\bar{f}}_{1,2}\left|+\right|{\bar{f}}_{3,2}\left|)\right|x_{2}|\right]{∥x∥}^{2}+2∥u∥∥B^{T}∥∥P∥∥x∥.$$

Concisely, Equation (72) can be expressed as:

(73) $$\dot{V}\leq -\lambda_{min}{\left(Q\right)∥x∥}^{2}+2|\bar{F}{\left(x\right)|∥x∥}^{2}+2∥u∥∥B^{T}∥∥P∥∥x∥$$

where

(74) $$\begin{array}{cc} \bar{F}\left(x\right) & =\left(|{\bar{f}}_{2,1}\left|+\right|{\bar{f}}_{3,1}|\right)|x_{1}|+\left(|{\bar{f}}_{1,2}\left|+\right|{\bar{f}}_{3,2}|\right)\left|x_{2}\right|\\ \; & =\underset{F^{{}^{\prime }}}{\underset{︸}{\left[\begin{array}{ccc} |{\bar{f}}_{2,1}\left|+\right|{\bar{f}}_{3,1}| & |{\bar{f}}_{1,2}\left|+\right|{\bar{f}}_{3,2}| & 0 \end{array}\right]}}\underset{x^{{}^{\prime }}}{\underset{︸}{\left[\begin{array}{c} |x_{1}|\\ |x_{2}|\\ |x_{3}| \end{array}\right]}}=F{}^{\prime }x{}^{\prime } \end{array}$$

Then, using the fact that $∥x{}^{\prime }∥=∥x∥$, yields

(75) $$\begin{array}{cc} \dot{V} & \leq -\left(\lambda_{min}\left(Q\right)-2\left|\bar{F}\left(x\right)\right|\right){∥x∥}^{2}+2∥u∥∥B^{T}∥∥P∥∥x∥\\ \; & \leq -\left(\lambda_{min}\left(Q\right)-2∥F{}^{\prime }∥∥x{}^{\prime }∥\right){∥x∥}^{2}+2∥u∥∥B^{T}∥∥P∥∥x∥\\ \; & \leq -\left(\lambda_{min}\left(Q\right)-2∥F{}^{\prime }∥∥x∥\right){∥x∥}^{2}+2∥u∥∥B^{T}∥∥P∥∥x∥ \end{array}$$

Notice that for a given δ, there exists some r>0 such that

(76) $$|\bar{F}\left(x\right)|<\delta ,\;\forall ∥x∥<r$$

Equation (75) can be rewritten as

(77) $$\begin{array}{cc} \dot{V}\leq -\left(1-\theta \right)\left[\lambda_{min}\left(Q\right)-2\delta \right]{∥x∥}^{2}- & [\theta \left(\lambda_{min}\left(Q\right)-2\delta \right){∥x∥}^{2}\\ \; & -2|u|∥B∥∥P∥∥x∥]\;\forall \;0<\theta <1 \end{array}$$

Then,

(78) $$\dot{V}\leq -\left(1-\theta \right)\left[\lambda_{min}\left(Q\right)-2\delta \right]{∥x∥}^{2}$$

for

(79) $$\delta <\frac{\lambda_{min}\left(Q\right)}{2}$$

and

$$∥x∥>\frac{2|u|∥B^{T}∥∥P∥}{\theta \left[\lambda_{min}\left(Q\right)-2\delta \right]}.$$

As a consequence, the system with closed-loop dynamics defined by Equation (44) is ISS with

$$W_{3}=\left(1-\theta \right)\left[\lambda_{min}\left(Q\right)-2\delta \right],$$

$$\rho =\frac{2|u|∥B^{T}∥∥P∥}{\theta \left[\lambda_{min}\left(Q\right)-2\delta \right]},$$

and

$$\gamma =\frac{\lambda_{max}\left(P\right)}{\lambda_{min}\left(P\right)}\frac{2|u|∥B^{T}∥∥P∥}{\theta \left[\lambda_{min}\left(Q\right)-2\delta \right]}.$$

### 5.4. IOS Analysis

The proof of the IOS property of the same system (represented by (44)) is based on Theorem 2 [29]. Consider the system

(80) $$\dot{x}=f\left(x,u\right),\;x\left(0\right)=x_{0}$$

(81) y=h(x)

According to Theorem 2, if the system (80) and (81) satisfies the conditions shown below, then the system is small-signal finite-gain $L_{p}$-stable for each p∈[1,∞]. In particular, for each $u\in L_{pe}$ with $sup_{0\leq t\leq \tau }∥u\left(t\right)∥\leq min\left\{r_{u},c_{1}c_{3}r/\left(c_{2}c_{4}L\right)\right\}$, the output satisfies

(82) $$∥y_{\tau }{∥}_{L_{p}}\leq \gamma {∥u_{\tau }∥}_{L_{p}}+\beta$$

where the parameters γ and β are given by

(83) $$\gamma =\eta_{2}+\frac{\eta_{1}c_{2}c_{4}}{c_{1}c_{3}},$$

and

(84) $$\beta =\eta_{1}∥x_{0}∥\sqrt{\frac{c_{2}}{c_{1}}}\rho ,\;where\;\rho =\{\begin{array}{cc} 1 & if\;p=\infty \\ {\left(\frac{2c_{2}}{c_{3}p}\right)}^{\left(1/p\right)} & if\;p\in [1,\infty ) \end{array}.$$

The parameters $c_{1}$, $c_{2}$, $c_{3}$, $c_{4}$, $\eta_{1}$, and $\eta_{2}$ are determined from inequalities in Theorem 2.

For the particular case of the system under consideration, Equations (80) and (81) are given as

(85) $$\dot{x}=f\left(x\right)=Ax+G\left(x\right)x+Bu;\;x\left(0\right)=x_{0}$$

(86) y=h(x)=Hx

where

(87) $$H=\left[\begin{array}{ccc} 0 & 0 & 1 \end{array}\right]$$

Thus, considering the Lyapunov function defined by Equation (63) yields the following sequence of partial results.

- From inequality (53)
  (88) $$\lambda_{min}{\left(P\right)∥x∥}^{2}\leq x^{T}Px\leq \lambda_{max}\left(P\right){∥x∥}^{2}$$
  Thus,
  $c_{1}=\lambda_{min}\left(P\right)$ and $c_{2}=\lambda_{max}\left(P\right)$
- From inequality (54)
  (89) $$\frac{\partial V}{\partial x}f\left(x,0\right)\leq -\left[\lambda_{min}\left(Q\right)-2\delta \right]{∥x∥}^{2}$$
  and therefore

$c_{3}=\left[\lambda_{min}\left(Q\right)-2\delta \right]$

- From inequality (55)
  (90) $$∥\frac{\partial V}{\partial x}∥=2∥x^{T}P∥\leq 2\lambda_{max}\left(P\right)∥x∥$$
  Thus, $c_{4}=2\lambda_{max}\left(P\right)$
- From inequality (56)
  (91) ∥f(t,x,u)−f(t,x,0)∥=∥Bu∥≤∥B∥∥u∥
  and therefore $L=∥B∥=\sqrt{\lambda_{max}\left(B^{T}B\right)}$.
- From inequality (57)
  (92) ∥h(x)∥≤∥H∥∥x∥,
  we obtain that
  $\eta_{1}=∥H∥=\sqrt{\lambda_{max}(H^{T}H})$ and $\eta_{2}=0$

From the above, the parameters $\gamma_{f}=\gamma$ and $\beta_{f}=\beta$ for the Medusa model are given by

(93) $$\gamma_{f}=\frac{\eta_{1}c_{2}c_{4}}{c_{1}c_{3}}=\frac{∥H∥\lambda_{max}^{2}\left(P\right)2\sqrt{\lambda_{max}\left(B^{T}B\right)}}{\lambda_{min}\left(P\right)\left[\lambda_{min}\left(Q\right)-2\delta \right]}$$

(94) $$\beta_{f}=\eta_{1}∥x_{0}∥\sqrt{\frac{c_{2}}{c_{1}}}\rho =∥H∥∥x_{0}∥\sqrt{\frac{\lambda_{max(P)}}{\lambda_{min}\left(P\right)}}\;for\;p=\infty$$

**Remark** **1.** *From a mathematical standpoint, the machinery adopted for the description of the systems under study, that is, inner (dynamic) and outer (kinematic) dynamics, is rooted in their characterization as ISS (input-to-state stable) or IOS (input-to-output stable) systems. This allows us to use powerful tools of nonlinear stability analysis. The core mathematical characterization of IOS and ISS hinges on the assumption that all functions involved in the description of the systems of interest are piecewise continuous in time and locally Lipschitz in the state and input variables. This is clearly indicated in the results on ISS described in Section 5.1 and Section 5.2, as applied to the system described by Equation *(44)*. Clearly, all functions involved (which capture the physical description of the vehicle) satisfy the conditions stated above. Identical comments apply to the results on IOS described in Section 5.3 and Section 5.4, as applied to the system described by Equations *(85)–(87)*, consisting of Equation *(44)* together with the trivial output function h(x)=Hx, H=[001] described in Equations (86) and (87). Again, all functions involved satisfy the conditions stated above (see Equation *(56)*. In addition, h(x) satisfies the extra output-related conditions in Equation *(57)*.*

## 6. Path Following Problem

Equipped with the above mathematical definitions and results, we now tackle the problem of path following. In the current setup, we design the kinematic (outer) loop without considering the dynamic (inner) loop or by assuming the inner loop is infinitely fast, which is not valid in practice. Moreover, the characteristics of inner loop controllers (such as heading and speed controllers) for many vehicles are provided in very general terms by their vendors. An example of these characteristics in a linear case is the approximate bandwidth and input-to-output stability gain (IOS) in the case of a nonlinear system [29]. Therefore, it is required for the system engineers to design or tune the outer-loop controller by considering these characteristics, such that the overall combined system is stable with the desired performance. However, this step necessitates going beyond qualitative assertions about the fast-slow temporal scale separation and quantitatively evaluating the combined inner-outer loop to obtain a relationship for assessing the combined system’s stability. The methods used are based on nonlinear control theory, wherein the cascade and feedback systems of interest are characterized in terms of their IOS properties. We use the IOS small-gain theorem to obtain quantitative relationships for best controller tuning applicable to a broad range of marine vehicles. 

The path-following controller discussed in this article is designed at the kinematic outer-loop that commands the inner-loop with the desired heading angles while the vehicle moves at an approximately constant speed. The idea is to provide a seamless implementation of the path-following control algorithms on the heterogeneous vehicles, which are pre-equipped with heading autopilots. To this effect, we assume that the heading control system is characterized only in terms of an IOS-like relationship without knowing detailed vehicle dynamic parameters.

### 6.1. Path Following: Straight Lines Problem

**Figure 5:**
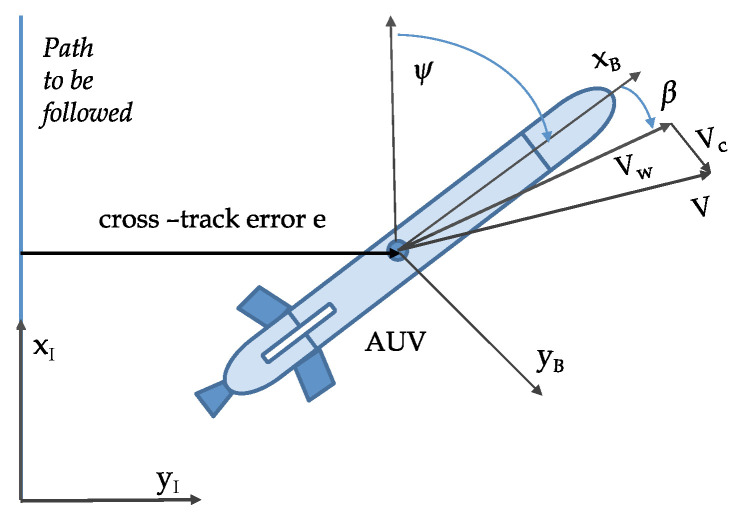
Marine vehicle body reference frame showing the cross-track error.

Figure 5 shows the path following problem for straight lines. In the figure, $\left\{I\right\}=\left\{x_{I},y_{I}\right\}$ represents the inertial reference frame, and $\left\{B\right\}=\left\{x_{B},y_{B}\right\}$ denotes a body reference frame fixed to the vehicle. Let us denote the position of the vehicle as vector *P* expressed in {I}. We assume that the ocean current velocity represented by $V_{c}$ expressed in {I} is constant. The velocity of the vehicle expressed in {I} is given by

$$\begin{array}{cc} \dot{P} & =R\left(\psi \right)V_{w}+V_{c}, \end{array}$$

where ψ is the yaw angle, $V_{w}$ denotes the velocity of the vehicle with respect to the water expressed in {B}, and R(.) is the rotation matrix from {B} to {I}, parameterized by ψ. Equivalently,

$$\begin{array}{cc} \dot{P} & =R\left(\psi +\beta \right){\left[∥V_{w}∥\;0\right]}^{T}+V_{c}, \end{array}$$

where β is the sideslip angle. Without any loss of generality, the straight-line path to be followed can be assumed to be along the *x*-axis of the inertial reference frame {I}. The evolution of the cross-track error *e* is given by

$$\begin{array}{cc} \dot{e} & =\sin\left(\psi +\beta \right)∥V_{w}∥+v_{cy}, \end{array}$$

where $v_{cy}$ denotes the component of $V_{c}$ along the unit vector $y_{I}$. The total speed of the vehicle is set by an equivalent speed of rotation of the stern propeller(s) and the heading of the vehicle is controlled either by differential mode of two stern propellers or by the stern rudders operated in common mode.

We assume that the total speed $∥V_{w}∥=U>∥V_{c}∥$ is constant. The objective is to command the heading angle which the vehicle can follow to drive the *e* to zero. In the following section, as a first step, we design an outer-loop controller at the kinematic level and show the convergence of the cross-track error to zero. In the second step, we include the yaw control dynamics (inner-loop) and determine the conditions and outer-loop tuning rules such that the complete inner-outer loop system is stable.

### 6.2. Path-Following Algorithm

To explain the rationale for the control law, we simplify the case by considering zero sideslip angle (this assumption will be lifted afterwards). In this case, the error dynamics are given by

(95) $$\begin{array}{cc} \dot{e} & =U\sin\left(\psi \right)+v_{cy}. \end{array}$$

If we consider $v_{cy}$ to be zero, then (95) can be re-written as

$$\begin{array}{cc} \dot{e} & =Uu, \end{array}$$

with u=sin(ψ). The choice of the control law $u=-(K_{1}/U)e$ would now ensure that *e* converges asymptotically and exponentially to the origin. In order to compensate for a fixed ocean current (bias) $v_{cy}$, an integral term is introduced in the virtual input *u*, which is now re-rewritten as

$$\begin{array}{cc} u & =-\frac{1}{U}\left(K_{1}e+K_{2}\int_{0}^{t}e(\tau )\;d\tau \right). \end{array}$$

As a consequence, the dynamics of *e* become

$$\begin{array}{cc} \dot{e}+K_{1}e+K_{2}\int_{0}^{t}e(\tau )\;d\tau = & 0 \end{array}$$

Let

$$\begin{array}{cc} \varsigma & =\int_{0}^{t}e(\tau )\;d\tau . \end{array}$$

Then,

(96) $$\begin{array}{cc} \ddot{\varsigma }+K_{1}\dot{\varsigma }+K_{2}\varsigma & =0 \end{array}$$

**Figure 6:**
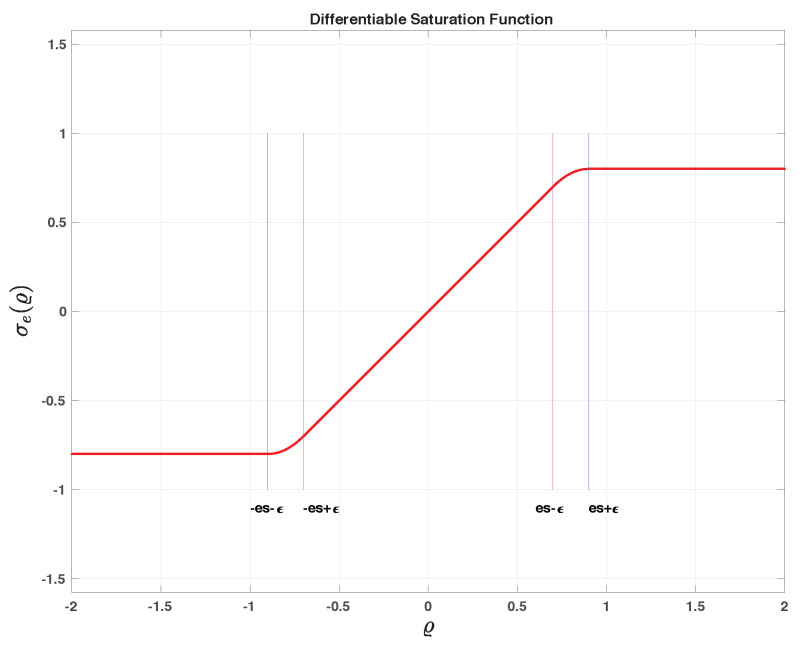
Differentiable saturation function.

The gains $K_{1}$ and $K_{2}$ can now be chosen so as to obtain a desired natural frequency and a desired damping factor in the above second-order system. The desired heading command obtained from the above virtual control is written as

$$\begin{array}{cc} \psi_{d} & =\sin^{-1}\left(\sigma_{e}\left(u\right)\right), \end{array}$$

where $\sigma_{e}$ is a differentiable saturation function [42] bounded between $\pm e_{s}$ with $0<e_{s}<1$, defined as

(97) $$\begin{array}{c} \sigma_{e}\left(\varrho \right)=\left\{\begin{array}{cc} \varrho & \mathrm{if}\;|\varrho |<e_{s}-\epsilon \\ +e_{s} & \mathrm{if}\;\varrho >e_{s}+\epsilon \\ -e_{s} & \mathrm{if}\;\varrho <-e_{s}-\epsilon \\ p_{1}\left(\varrho \right)=-c_{1}\varrho^{2}+c_{2}\varrho -c_{3} & \mathrm{if}\;\varrho \in \;]e_{s}-\epsilon ,e_{s}+\epsilon ]\\ p_{2}\left(\varrho \right)=c_{1}\varrho^{2}+c_{2}\varrho +c_{3} & \mathrm{if}\;\varrho \in \;[-e_{s}-\epsilon ,-e_{s}+\epsilon [ \end{array}\right. \end{array}$$

where $0<\epsilon <e_{s}$ can be arbitrarily small, with $c_{1}=\frac{1}{4\epsilon }$, $c_{2}=\frac{1}{2}+\frac{e_{s}}{2\epsilon }$, and $c_{3}=\frac{\epsilon^{2}-2\epsilon e_{s}+e_{s}^{2}}{4\epsilon }$. The saturation function is introduced to guarantee that the argument of $sin^{-1}\left(.\right)$ lies in the interval [−1,+1], see Figure 6.

With the introduction of integrator in the control law, it is important to have an anti-windup mechanism in the integral term of *u*. Thus, the final form of the control law for $\psi_{d}$ involves a new definition of *u* and is given in terms of the operator $f:e\to \psi_{d}$ defined by

(98) $$\begin{array}{cc} \psi_{d} & =\sin^{-1}\left(\sigma_{e}\left(u\right)\right);u=\left(-\frac{K_{1}e}{U}-\frac{K_{2}}{U}\varsigma \right) \end{array}$$

where ς is the output of the dynamical system $f_{aw}\left(e\right):e\to \varsigma$ with realization

(99) $$\begin{array}{cc} \dot{\varsigma } & =e+K_{a}\left[-\frac{K_{1}e}{U}-\frac{K_{2}\varsigma }{U}-\sigma_{e}\left(-\frac{K_{1}e}{U}-\frac{K_{2}\varsigma }{U}\right)\right], \end{array}$$

and $K_{a}$ is an anti-windup gain to control the integrator’s charge and discharge rate. In what follows, we show with the help of Lyapunov-based analysis tools that using the above control law, the cross-track error converges to zero if the actual vehicle heading ψ equals $\psi_{d}$.

### 6.3. Convergence of Cross-Track Error without the Inner Loop Dynamics

Using the control law mentioned in (98) and (99), and $K_{a}=\frac{U}{K_{1}}$, the closed-loop kinematic equations can now be written as,

(100) $$\begin{array}{cc} \dot{e} & =U\sigma_{e}\left(-\frac{K_{1}e}{U}-\frac{K_{2}\varsigma }{U}\right)+V_{yc} \end{array}$$

(101) $$\begin{array}{cc} \dot{\varsigma } & =-\frac{K_{2}}{K_{1}}\varsigma -\frac{U}{K_{1}}\sigma_{e}\left(-\frac{K_{1}e}{U}-\frac{K_{2}\varsigma }{U}\right), \end{array}$$

Define the new set of variables

(102) $$\begin{array}{cc} x_{1} & =e\\ x_{2} & =\varsigma -K, \end{array}$$

where *K* is a constant.

The equations of motion can then be written as

(103) $$\begin{array}{cc} {\dot{x}}_{1} & =U\sigma_{e}\left(-\frac{K_{1}x_{1}}{U}-\frac{K_{2}x_{2}}{U}-\frac{KK_{2}}{U}\right)+V_{yc} \end{array}$$

(104) $$\begin{array}{cc} {\dot{x}}_{2} & =-\frac{K_{2}}{K_{1}}x_{2}-\frac{K_{2}}{K_{1}}K-\frac{U}{K_{1}}\sigma_{e}\left(-\frac{K_{1}x_{1}}{U}-\frac{K_{2}x_{2}}{U}-\frac{KK_{2}}{U}\right). \end{array}$$

We define another set of variables as

(105) $$\begin{array}{cc} rly_{1} & =\frac{K_{1}x_{1}}{U}+\frac{K_{2}x_{2}}{U}r\\ y_{2} & =\frac{K_{2}x_{2}}{U}, \end{array}$$

In terms of the new variables above,

(106) $$\begin{array}{cc} {\dot{y}}_{1} & =-K_{1}\sigma_{e}\left(y_{1}+\frac{KK_{2}}{U}\right)+K_{1}\frac{V_{yc}}{U}+{\dot{y}}_{2}\\ {\dot{y}}_{2} & =-\frac{K_{2}}{K_{1}}y_{2}-\frac{K_{2}}{K_{1}}\frac{KK_{2}}{U}+\frac{K_{2}}{K_{1}}\sigma_{e}\left(y_{1}+\frac{KK_{2}}{U}\right), \end{array}$$

At this point, we explore an important property of the $\sigma_{e}$ function defined in (97).

**Property** **1.**
$$\begin{array}{cc} \sigma_{e}\left(Z+x\right)-Z & =\sigma_{l_{s}}^{u_{s}}\left(x\right)\;\;\forall \;\;\left|Z\right|<e_{s}, \end{array}$$

*where $l_{s}=-e_{s}-\left|Z\right|$ and $u_{s}=e_{s}-\left|Z\right|$.*

To simplify the notation, we use σ instead of $\sigma_{l_{s}}^{u_{s}}$ from here on. Using this property, we can write

(107) $$\begin{array}{c} \sigma_{e}\left(y_{1}+\frac{KK_{2}}{U}\right)-\frac{KK_{2}}{U}=\sigma \left(y_{1}\right),\;\;\forall \;\;\left|\frac{KK_{2}}{U}\right|<e_{s}, \end{array}$$

and later in the proof, it will be evident that $|\frac{KK_{2}}{U}|<e_{s}$. Thus, by simplifying further, we get

(108) $$\begin{array}{cc} {\dot{y}}_{1} & =-K_{1}\sigma \left(y_{1}\right)-K_{1}\frac{KK_{2}}{U}+K_{1}\frac{V_{yc}}{U}+{\dot{y}}_{2}\\ {\dot{y}}_{2} & =\frac{K_{2}}{K_{1}}\sigma \left(y_{1}\right)-\frac{K_{2}}{K_{1}}y_{2} \end{array}$$

Choosing $V\left(y_{1},y_{2}\right)=\int_{0}^{y_{1}}\sigma \left(\eta \right)d\eta +\frac{1}{2}y_{2}^{2}$ as a Lyapunov candidate function yields

$\begin{array}{c} \begin{array}{cc} \dot{V} & =\sigma \left(y_{1}\right){\dot{y}}_{1}+y_{2}{\dot{y}}_{2}\\ \; & =\sigma \left(y_{1}\right)\left[-K_{1}\sigma \left(y_{1}\right)-K_{1}\frac{KK_{2}}{U}+\right.\\ \; & \;\left.K_{1}\frac{V_{yc}}{U}+\frac{K_{2}}{K_{1}}\sigma \left(y_{1}\right)-\frac{K_{2}}{K_{1}}y_{2}\right]\\ \; & =-\left(K_{1}-\frac{K_{2}}{K_{1}}\right)\sigma^{2}\left(y_{1}\right)-\frac{K_{2}}{K_{1}}y_{2}^{2}+\\ \; & \;\sigma \left(y_{1}\right)\left[K_{1}\frac{V_{yc}}{U}-K_{1}\frac{KK_{2}}{U}\right]. \end{array} \end{array}$

Making

(109) $$\begin{array}{c} K=\frac{V_{yc}}{K_{2}} \end{array}$$

yields

(110) $$\begin{array}{cc} \dot{V} & =-\left(K_{1}-\frac{K_{2}}{K_{1}}\right)\sigma^{2}\left(y_{1}\right)-\frac{K_{2}}{K_{1}}y_{2}^{2} \end{array}$$

At this point, it is reasonable to assume that the vehicle speed with respect to water is larger than the intensity of the ocean current, that is,

(111) $$\begin{array}{c} U>\frac{1}{e_{s}}∥V_{c}∥. \end{array}$$

Using the above assumption, it is now straightforward to show that $|\frac{KK_{2}}{U}|<e_{s}$.

Thus,

(112) $$\begin{array}{c} \dot{V}<0\;\forall \;K_{1}>\frac{K_{2}}{K_{1}}. \end{array}$$

We therefore conclude that the origin $y_{1}=y_{2}=0$ is asymptotically stable. It is now trivial to show that the cross-track error *e* will tend to zero and the integrator ς will charge up to $\frac{V_{yc}}{K_{2}}$, in order to “learn” the currents as time increases.

### 6.4. Inner-Loop Dynamics

**Figure 7:**
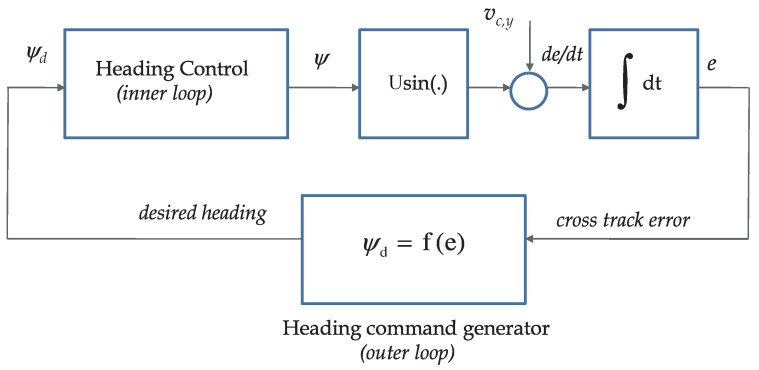
Path-following controller with two-scale inner-outer loop approach.

The key goal of this paper is to show that “identical behavior” is obtained when the dynamics of the heading autopilot (inner loop) and the sideslip of the vehicle are taken into account. In particular, we show that *the basic structure and the simplicity of the outer-loop control law are preserved*. The theoretical machinery used to prove stability borrows from IOS concepts and a related small-gain theorem. See [29] for a fast-paced introduction to the subject and [43,44] for interesting applications of control techniques that bear affinity with inner-outer loop control structures. Here, we indicate briefly how the existence of the heading autopilot is taken into account without having to change the structure of the outer-loop described before. The resulting control scheme is depicted in Figure 7, where the heading autopilot plays the role of an inner loop.

This section addresses explicitly the inclusion of the inner-loop dynamics, thus lifting the assumption that the actual heading ψ equals the desired heading $\psi_{d}$. Let

$$\begin{array}{cc} \tilde{\psi } & =\psi -\psi_{d} \end{array}$$

be the mismatch between actual and desired heading angles. We assume that the autopilot characteristics can be described in very general terms as an IOS system, see [29]. In order to understand the rationale for this characterization, notice that if the inner-loop dynamics are linear with static gain equal to 1, then its dynamics admit a realization of the form

$$\begin{array}{cc} \dot{x} & =Ax+B\psi_{d}\\ \psi & =Cx \end{array}$$

with $CA^{-1}B=1$. In this case, the coordinate transformation $\eta =x+A^{-1}B\psi_{d}$ yields the realization

$$\begin{array}{cc} \dot{\eta } & =A\eta +A^{-1}B{\dot{\psi }}_{d}\\ \tilde{y} & =C\eta \end{array}$$

for the operator from $\dot{\psi }$ to $\tilde{y}$, with $\tilde{y}=\tilde{\psi }+\beta$ that characterizes the inner-loop dynamics, where β is sideslip angle and the output $\tilde{y}$ is the sum of the heading angle and sideslip angle. An IOS characterization of the loop can be easily derived from the above system matrices [29]. Notice, however, that this type of description applies also to general nonlinear systems of the form

$$\begin{array}{cc} \dot{\eta } & =g(\eta ,{\dot{\psi }}_{d})\\ \tilde{y} & =h(\eta ,{\dot{\psi }}_{d}) \end{array}$$

and allows for a somewhat loose, yet quantifiable description of the inner-loop dynamics. This justifies the IOS characterization of the inner loop dynamics as

(113) $$\begin{array}{cc} ∥\tilde{y}\left(t\right)∥ & \leq \gamma_{f}∥\dot{\psi_{d}}\left(t\right)∥+\beta_{f}, \end{array}$$

where $\gamma_{f}$ and $\beta_{f}$ are nonnegative constants. The above characterization captures in a rigorous mathematical framework simple physical facts about the inner-loop control system. Namely, (i) if the time-derivative of the heading reference $\psi_{d}$ is bounded, then the heading-tracking error is bounded and (ii) the dynamics of the inner-loop system can be characterized in terms of bandwidth-like characteristics that are reflected in $\beta_{f}$ and $\gamma_{f}$, see [29]. A simple exercise with a first-order system will convince the reader that as the bandwidth of the system increases, $\gamma_{f}$ will decrease. For practical purposes, the latter can be viewed as a “tuning knob” during the path-following controller design phase. For analysis purposes, it is also required to ensure that not only $\tilde{y}$ but also the remaining variables in the inner loop be bounded in response to ${\dot{\psi }}_{d}$. This fact can be easily captured with an ISS condition of the type

(114) $$\begin{array}{cc} ∥\eta (t)∥ & \leq \beta_{g}\left(∥\eta (0)∥,t\right)+\gamma_{g}\left(\underset{t_{0}\leq \tau \leq t}{\sup}∥\dot{\psi_{d}}\left(\tau \right)∥\right), \end{array}$$

for some $\beta_{g}\in \mathrm{KL}$ and $\gamma_{g}\in K$. We have shown before that such a condition holds. At this point, we make the key observation that the complete path-following control system can be represented as the interconnected structure depicted in Figure 8. The latter can be further abstracted to the scheme in Figure 9 consisting of blocks $H_{1}:\tilde{y}\to {\dot{\psi }}_{d}$ and $H_{2}:{\dot{\psi }}_{d}\to \tilde{y}$, a description of which is given next. To this effect, using the control law mentioned in (98) and (99), the system $H_{1}$ clearly admits the following representation

(115) $$\begin{array}{cc} \dot{e} & =U\sin\left(\tilde{y}+\psi_{d}\right)+v_{cy},\\ \dot{\varsigma } & =-\frac{K_{2}}{K_{1}}\varsigma -\frac{U}{K_{1}}\sigma_{e}\left(-\frac{K_{1}e}{U}-\frac{K_{2}\varsigma }{U}\right)\\ \psi_{d} & =\sin^{-1}\left(\sigma_{e}\left(-\frac{K_{1}e}{U}-\frac{K_{2}}{U}\varsigma \right)\right), \end{array}$$

and $H_{2}$ satisfies the IOS stability condition in (113).

### 6.5. Convergence: Realistic Inner-Loop Dynamics

The proof that $H_{1}$ is IOS hinges on the facts that $H_{1}$ is the composition of two auxiliary systems $H_{a1}:\tilde{y}\to e$ and $H_{a2}:e\to \dot{\psi }$ and that both are IOS. This is done next. Expanding Equation (115) and following the transformation of variables as mentioned in (105), the equation of motion can be rewritten as

(116) $$\begin{array}{cc} {\dot{y}}_{1} & =-K_{1}\cos\tilde{y}\sigma_{e}\left(y_{1}+\frac{KK_{2}}{U}\right)+K_{1}\sin\tilde{y}\cos\psi_{d}\;+\\ \; & \;\frac{K_{1}}{U}V_{yc}+{\dot{y}}_{2}\\ {\dot{y}}_{2} & =\frac{K_{2}}{K_{1}}\left[\sigma_{e}\left(y_{1}+\frac{KK_{2}}{U}\right)-\frac{KK_{2}}{U}-\frac{K_{2}}{K_{1}}y_{2}\right], \end{array}$$

By adding and subtracting the term $K_{1}\cos\tilde{y}\frac{KK_{2}}{U}$, and by using the special property of the function $\sigma_{e}$ in (107), we can write

(117) $$\begin{array}{c} \begin{array}{cc} {\dot{y}}_{1} & =-K_{1}\cos\tilde{y}\sigma \left(y_{1}\right)+K_{1}\sin\tilde{y}\cos\psi_{d}+\frac{K_{1}}{U}V_{yc}\\ \; & \;-K_{1}\cos\tilde{y}\frac{KK_{2}}{U}+\frac{K_{2}}{K_{1}}\sigma \left(y_{1}\right)-\frac{K_{2}}{K_{1}}y_{2}\\ {\dot{y}}_{2} & =\frac{K_{2}}{K_{1}}\sigma \left(y_{1}\right)-\frac{K_{2}}{K_{1}}y_{2} \end{array} \end{array}$$

Choosing the same Lyapunov function as in Section 6.3 yields

(118) $$\begin{array}{c} \begin{array}{cc} \dot{V} & =\sigma \left(y_{1}\right){\dot{y}}_{1}+y_{2}{\dot{y}}_{2}\\ \; & =\sigma \left(y_{1}\right)\left[-K_{1}\cos\tilde{y}\sigma \left(y_{1}\right)+K_{1}\sin\tilde{y}\cos\psi_{d}\right.\\ \; & \left.\;+\frac{K_{1}}{U}V_{yc}-K_{1}\cos\tilde{y}\frac{KK_{2}}{U}+\frac{K_{2}}{K_{1}}\sigma \left(y_{1}\right)-\frac{K_{2}}{K_{1}}y_{2}\right]\\ \; & \;+y_{2}\left[\frac{K_{2}}{K_{1}}\sigma \left(y_{1}\right)-\frac{K_{2}}{K_{1}}y_{2}\right]\\ \; & =-K_{1}\cos\tilde{y}\sigma^{2}\left(y_{1}\right)-\frac{K_{2}}{K_{1}}y_{2}^{2}\\ \; & \;+\sigma \left(y_{1}\right)\left[K_{1}\sin\tilde{y}\cos\psi_{d}+\frac{K_{1}}{U}V_{yc}-K_{1}\cos\tilde{y}\frac{KK_{2}}{U}\right]\\ \; & \;+\frac{K_{2}}{K_{1}}\sigma^{2}\left(y_{1}\right)\\ \; & =-\left(K_{1}\cos\tilde{y}-\frac{K_{2}}{K_{1}}\right)\sigma^{2}\left(y_{1}\right)-\frac{K_{2}}{K_{1}}y_{2}^{2}\\ \; & \;+\sigma \left(y_{1}\right)\left[K_{1}\sin\tilde{y}\cos\psi_{d}+\frac{K_{1}}{U}V_{yc}-K_{1}\cos\tilde{y}\frac{KK_{2}}{U}\right], \end{array} \end{array}$$

Now, substituting *K* from (109), we obtain

$\begin{array}{c} \begin{array}{cc} \dot{V} & =-\left(K_{1}\cos\tilde{y}-\frac{K_{2}}{K_{1}}\right)\sigma^{2}\left(y_{1}\right)-\frac{K_{2}}{K_{1}}y_{2}^{2}\\ \; & \;+\sigma \left(y_{1}\right)\left[K_{1}\sin\tilde{y}\cos\psi_{d}+\frac{K_{1}}{U}V_{yc}\left(1-\cos\tilde{y}\right)\right]. \end{array} \end{array}$

For

(119) $$\begin{array}{c} K_{1}\cos\tilde{y}\geq \frac{K_{2}}{K_{1}}+\delta ,\;\mathrm{with}\;0<\delta \leq K_{1}-\frac{K_{2}}{K_{1}}, \end{array}$$

we can further simplify the equations as

$$\begin{array}{c} \begin{array}{cc} \dot{V} & \leq -\delta \sigma^{2}\left(y_{1}\right)-\frac{K_{2}}{K_{1}}y_{2}^{2}\\ \; & \;+\sigma \left(y_{1}\right)\left[K_{1}\sin\tilde{y}\cos\psi_{d}+\frac{K_{1}}{U}V_{yc}\left(1-\cos\tilde{y}\right)\right],\\ \; & =-\delta \sigma^{2}\left(y_{1}\right)-\frac{K_{2}}{K_{1}}y_{2}^{2}\\ \; & \;+|\sigma \left(y_{1}\right)|\left[|K_{1}\sin\tilde{y}\cos\psi_{d}\left|+\right|\frac{K_{1}}{U}V_{yc}\left|\right|1-\cos\tilde{y}|\right],\\ \; & =-\delta \sigma^{2}\left(y_{1}\right)-\frac{K_{2}}{K_{1}}y_{2}^{2}\\ \; & \;+|\sigma \left(y_{1}\right)|K_{1}\left[|\frac{V_{yc}}{U}\left|\right|1-\cos\tilde{y}\left|+\right|\sin\tilde{y}|\right],\\ \; & =-\delta \left(1-\theta \right)\sigma^{2}\left(y_{1}\right)-\delta \theta \sigma^{2}\left(y_{1}\right)-\frac{K_{2}}{K_{1}}y_{2}^{2}\\ \; & \;+|\sigma \left(y_{1}\right)|K_{1}\left[|\frac{V_{yc}}{U}\left|\right|1-\cos\tilde{y}\left|+\right|\sin\tilde{y}|\right], \end{array} \end{array}$$

where 0<θ<1.

This implies that

(120) $$\begin{array}{c} \begin{array}{cc} \dot{V} & <-\delta \left(1-\theta \right)\sigma^{2}\left(y_{1}\right)-\frac{K_{2}}{K_{1}}y_{2}^{2}\\ \; & \;\forall |\sigma \left(y_{1}\right)|>\frac{K_{1}}{\delta \theta }\left(|\frac{V_{yc}}{U}\left|\right|1-\cos\tilde{y}\left|+\right|\sin\tilde{y}|\right). \end{array} \end{array}$$

Since $\left(|\frac{V_{yc}}{U}\left|\right|1-\cos\tilde{y}\left|+\right|\sin\tilde{y}|\right)$ is bounded by $\left(|\frac{V_{yc}}{U}|+1\right)\left(|\tilde{y}|\right)$, it follows that

(121) $$\begin{array}{c} \dot{V}<0\;\forall |\sigma \left(y_{1}\right)|>\;\frac{K_{1}}{\delta \theta }\left(|\frac{V_{yc}}{U}|+1\right)\left|\tilde{y}\right| \end{array}$$

thus showing that $y={\left[y_{1}\;y_{2}\right]}^{T}$ is ISS with restriction given by (119) on the input. Thus, from the definition of ISS, we obtain

(122) $$\begin{array}{c} ∥y\left(t\right)∥\leq \beta_{l}\left(∥y(0)∥,t\right)+\gamma_{l}\left(\underset{0\leq \tau ⩽t}{\sup}\left|\tilde{y}\left(\tau \right)\right|\right)\;\;\forall \;t\geq 0, \end{array}$$

where $\beta_{l}$ is a class KL-function, and $\gamma_{l}$ is a class K-function. To show that $H_{1}:\tilde{y}\to {\dot{\psi }}_{d}$ is IOS, we start by computing ${\dot{\psi }}_{d}$. The control law $\psi_{d}$ is given by

(123) $$\begin{array}{cc} \psi_{d} & =\sin^{-1}\left\{\sigma_{e}\left(-\frac{K_{1}e}{U}-\frac{K_{2}}{U}\varsigma \right)\right\}. \end{array}$$

Using (105), the above can be written as

(124) $$\begin{array}{cc} \psi_{d} & =\sin^{-1}\left\{\sigma_{e}\left(-y_{1}-\frac{KK_{2}}{U}\right)\right\}. \end{array}$$

Defining $\xi =-y_{1}-\frac{KK_{2}}{U}$, the time derivative of $\psi_{d}$ is given by

(125) $$\begin{array}{cc} \frac{d\psi_{d}}{dt} & =\frac{d\psi_{d}}{d\sigma_{e}\left(\xi \right)}\;\frac{d\sigma_{e}\left(\xi \right)}{d\xi }\;\frac{d\xi }{dt}\\ \; & =-{\dot{y}}_{1}\frac{d}{d\xi }\sigma_{e}\left(\xi \right){\left[1-{\left\{\sigma_{e}\left(-y_{1}-\frac{KK_{2}}{U}\right)\right\}}^{2}\right]}^{-\frac{1}{2}} \end{array}$$

with

(126) $$\begin{array}{cc} {\left[1-{\left\{\sigma_{e}\left(-y_{1}-\frac{KK_{2}}{U}\right)\right\}}^{2}\right]}^{-\frac{1}{2}} & \leq \eta \end{array}$$

where $\eta =\frac{1}{{\left(1-e_{s}^{2}\right)}^{\frac{1}{2}}}.$

From the definition of $\sigma_{e}\left(.\right)$, $\frac{d}{d\xi }\sigma_{e}\left(\xi \right)$ is bounded by 1. Thus, ${\dot{\psi }}_{d}$ in (125) is bounded by

(127) $$\begin{array}{cc} |{\dot{\psi }}_{d}| & \leq \eta |{\dot{y}}_{1}|. \end{array}$$

Equipped with the above result and the one in (122), we will now show that the system $H_{1}$ is IOS.

From Equation (117), we have

$$\begin{array}{cc} {\dot{y}}_{1} & =-K_{1}\cos\tilde{y}\sigma \left(y_{1}\right)+K_{1}\sin\tilde{y}\cos\psi_{d}+\frac{K_{1}}{U}V_{yc}-\\ \; & \;K_{1}\cos\tilde{y}\frac{KK_{2}}{U}+\frac{K_{2}}{K_{1}}\sigma \left(y_{1}\right)-\frac{K_{2}}{K_{1}}y_{2}. \end{array}$$

With $K=\frac{V_{yc}}{K_{2}}$,

$$\begin{array}{cc} {\dot{y}}_{1} & =-K_{1}\cos\tilde{y}\sigma \left(y_{1}\right)+\frac{K_{2}}{K_{1}}\sigma \left(y_{1}\right)-\frac{K_{2}}{K_{1}}y_{2}+\\ \; & \;K_{1}\left[\sin\tilde{y}\cos\psi_{d}+\frac{V_{yc}}{U}\left(1-\cos\tilde{y}\right)\right]. \end{array}$$

Taking the absolute value of both sides yields

$$\begin{array}{cc} |{\dot{y}}_{1}| & \leq \left(K_{1}+\frac{K_{2}}{K_{1}}\right)|\sigma \left(y_{1}\right)|+\frac{K_{2}}{K_{1}}\left|y_{2}\right|+\\ \; & \;K_{1}\left[|\sin\tilde{y}|+\frac{V_{yc}}{U}\left|\left(1-\cos\tilde{y}\right)\right|\right]\\ |{\dot{y}}_{1}| & \leq \left(K_{1}+\frac{K_{2}}{K_{1}}\right)|\sigma \left(y_{1}\right)|+\frac{K_{2}}{K_{1}}|y_{2}|+K_{1}\left(1+\frac{V_{yc}}{U}\right)\left|\tilde{y}\right| \end{array}$$

Thus,

$$\begin{array}{cc} |{\dot{\psi }}_{d}| & \leq C_{1}|\sigma \left(y_{1}\right)|+C_{2}|y_{2}|+C_{3}\left|\tilde{y}\right| \end{array}$$

where $C_{1}=\eta \left(K_{1}+\frac{K_{2}}{K_{1}}\right),C_{2}=\eta \frac{K_{2}}{K_{1}},\;\mathrm{and}\;C_{3}=\eta K_{1}\left(1+\frac{V_{yc}}{U}\right)$. Using the fact $|y_{1}\left|+\right|y_{2}|={∥y∥}_{1}$ and $|\sigma (y_{1})\left|\leq \right|y_{1}|$, we can state that

$$\begin{array}{c} C_{1}|\sigma \left(y_{1}\right)|+C_{2}\left|y_{2}\right|\leq max\left(C_{1},C_{2}\right){∥y∥}_{1}\leq C_{1}{∥y∥}_{1}. \end{array}$$

Thus,

(128) $$\begin{array}{cc} |{\dot{\psi }}_{d}| & \leq \beta_{1}+\gamma_{1}\left|\tilde{y}\right| \end{array}$$

where $\beta_{1}=C_{1}\beta_{l}$ and $\gamma_{1}=C_{1}\gamma_{l}+C_{3}$ (using the conditions in Theorem 2 and Equation (121)) is given by

(129) $$\begin{array}{c} \gamma_{1}=\eta K_{1}\left(\frac{V_{yc}}{U}+1\right)\left[\frac{1}{\delta \theta }\left(K_{1}+\frac{K_{2}}{K_{1}}\right)+1\right], \end{array}$$

with $\gamma_{1}$ showing explicit dependence on $K_{1},K_{2}$. In conclusion, the systems $H_{1}$ and $H_{2}$ are both IOS. It can now be shown, using the small gain theorem in [29], that the interconnected system is stable if $\gamma_{1}\gamma_{f}<1$. This result yields a rule for the choice of gains $K_{1},K_{2}$ (as functions of the inner-loop dynamic parameters) so that stability is obtained. Hence, we show using a small gain theorem that the above interconnected system is closed-loop-stable and all signals are bounded.

*Notice that for restriction (119) to be feasible, it is important that $K_{1}>\frac{K_{2}}{K_{1}}$. In other words, if we choose $K_{1}=2\xi \omega_{n}$ and $K_{2}=\omega_{n}^{2}$, where ξ and $\omega_{n}$ are damping factor and natural frequency, respectively, then ξ>0.5 must be used as design parameter.*

### 6.6. An Example

Let us take a simple illustrative example, considering that the sideslip angle β is zero. In this situation, the inner Loop (Heading control) is characterized in terms of an IOS relationship given in (113) with $\tilde{y}=\tilde{\psi }$, where $\tilde{\psi }=\psi -\psi_{d}$, see (44). For such a system, it is straightforward to show that the system is finite-gain $L_{\infty }$-stable, that is,

(130) $$\begin{array}{cc} {∥\tilde{\psi }∥}_{\infty } & \leq \gamma_{f}{∥\dot{\psi_{d}}∥}_{\infty }+\beta_{f} \end{array}$$

with the gain $\gamma_{f}$ given by

(131) $$\begin{array}{cc} \gamma_{f} & =\frac{2\lambda_{max}^{2}\left(Q\right){∥A^{-1}B∥}_{2}{∥C∥}_{2}}{\lambda_{min}\left(Q\right)}, \end{array}$$

where *Q* is the solution of the Lyapunov equation $QA+A^{T}Q=-I$. Approximating the inner loop as a first-order system with dynamics given by

(132) $$\begin{array}{cc} \dot{\psi } & =-a\psi +a\psi_{d}. \end{array}$$

(133) $$\begin{array}{cc} y_{2} & =\psi \end{array}$$

it follows that

(134) $$\begin{array}{cc} \gamma_{f} & =\frac{2{\left(\frac{1}{2a}\right)}^{2}}{\left(\frac{1}{2a}\right)} \end{array}$$

(135) $$\begin{array}{cc} \gamma_{f} & =\frac{1}{a}, \end{array}$$

yielding the stability condition

(136) $$\begin{array}{cc} \gamma_{1} & <a. \end{array}$$

From (129), we have

(137) $$\begin{array}{cc} \eta K_{1}\left(\frac{V_{yc}}{U}+1\right)\left[\frac{1}{\delta \theta }\left(K_{1}+\frac{K_{2}}{K_{1}}\right)+1\right]\; & <a. \end{array}$$

For an inner-loop bandwidth a=1 rad/s, using the parameters mentioned in Table 1 and equating $\gamma_{1}$ to *a*, the natural frequency $\omega_{n}$ for the outer loop should not be more than 0.095 rad/s.

Thus, in terms of bandwidth-like characterization, the inner loop bandwidth should be approximately 10 times higher than outer-loop bandwidth. The parameters δ and θ chosen in Table 1 impose a restriction of $\tilde{\psi }<20.7$ degrees.

### 6.7. Relation between Outer-Loop Path Following and Using a Variable Look-Ahead Visibility Distance Line-of-Sight Guidance

**Figure 10:**
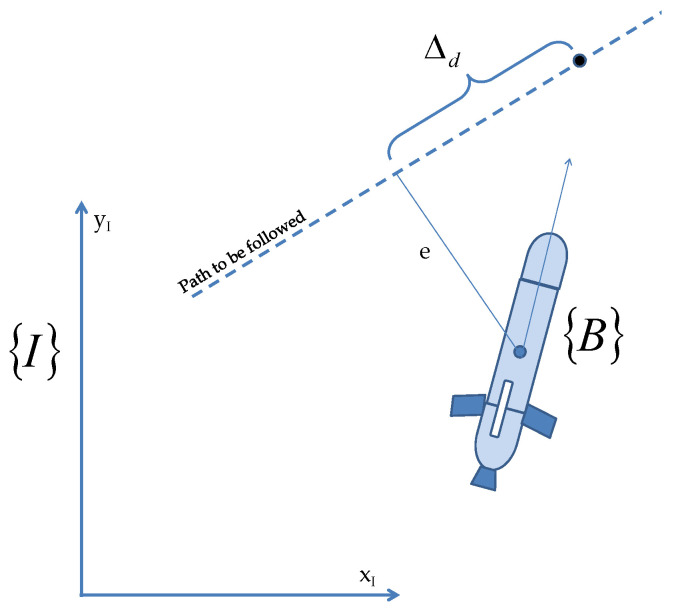
Line-of-sight guidance using *look-ahead distance*.

Consider a case of simple path-following controller without the integral term, such that the control law can be written as

(138) $$\begin{array}{c} \psi_{d}=sin^{-1}\left(\frac{-K_{1}e}{U}\right), \end{array}$$

whereas, in the case of *look-ahead distance* line-of-sight guidance, the control law is given by

(139) $$\begin{array}{c} \psi_{d}=tan^{-1}\left(-\frac{e}{\Delta_{d}}\right), \end{array}$$

where $\Delta_{d}$ is the *look-ahead distance* as shown in Figure 10.

By equating Equations (138) and (139), we get a relationship between gain $K_{1}$ and the *look-ahead distance* $\Delta_{d}$ as follows:

(140) $$\begin{array}{cc} -\frac{K_{1}e}{U} & =sin\left(tan^{-1}\left(-\frac{e}{\Delta_{d}}\right)\right) \end{array}$$

(141) $$\begin{array}{cc} K_{1} & =\frac{U}{{\left(\Delta_{d}^{2}+e^{2}\right)}^{\frac{1}{2}}} \end{array}$$

(142) $$\begin{array}{cc} \Rightarrow \Delta_{d} & =\pm \frac{1}{K_{1}}{\left(U^{2}-K_{1}^{2}e^{2}\right)}^{\frac{1}{2}} \end{array}$$

**Figure 11:**
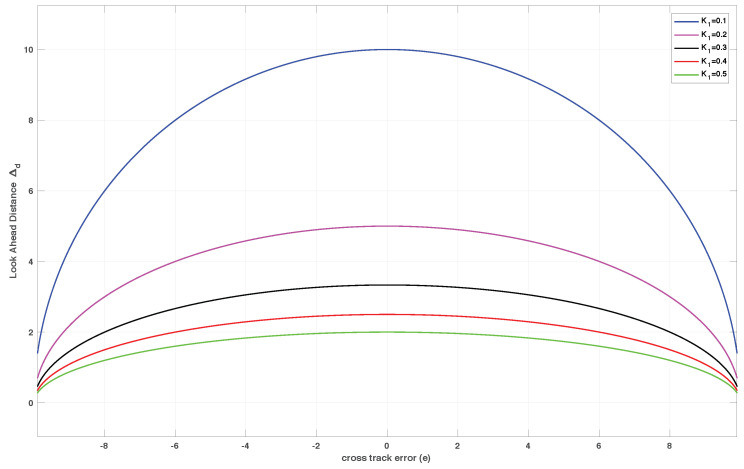
Look-ahead distance plotted against the cross-track error with different gains.

Figure 11 shows the variation of look-ahead distance with cross-track error at different bandwidth in the above example. Notice that the look-ahead distance is a function of cross-track error (not fixed in this setup), and increases as the gain $K_{1}$ is reduced.

## 7. Path Following Problem: Arcs

**Figure 12:**
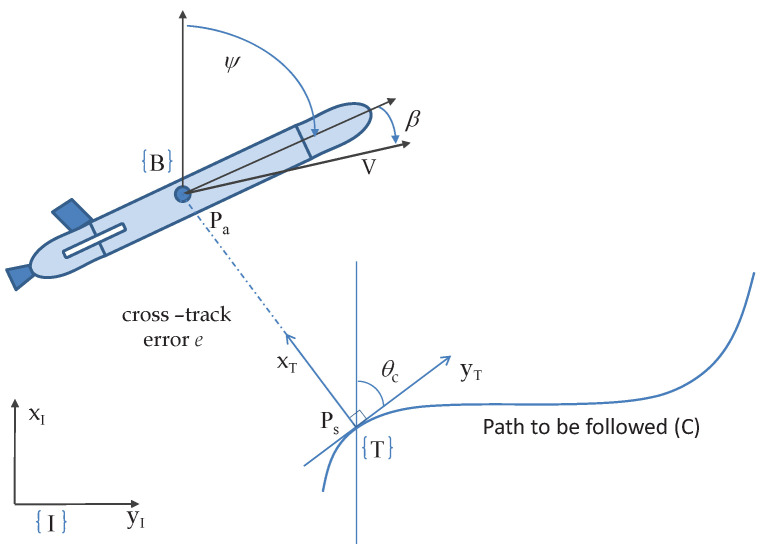
Cross-track error for straight-line following.

Let $P_{a}$ be the position of the vehicle (see Figure 12) in an inertial reference frame {I} and the associated Seret–Frenet frame *T* defined on the curve such that its origin $\left(P_{s}\right)$ is the orthogonal projection of the point $P_{a}$ onto curve; thus, *e* is the cross-track error with the coordinates of vehicle (0,e) in {T}. With *U* as the speed of the vehicle, the kinematic equation for the evolution of *e* is given by

(143) $$\begin{array}{cc} \dot{e} & =U\sin(\psi -\theta_{c}) \end{array}$$

where ψ is the vehicle orientation with respect to the {I}, $\theta_{c}$ is the angle of tangent at point $P_{s}$ measured from abscissa of {I}, and $R_{c}\left(P_{s}\right)$ is the radius of curvature at {T} (see [6]). Note that ${\dot{\theta }}_{c}$ becomes infinite when the $R_{c}\left(P_{s}\right)=e$, which in turn means that the vehicle is positioned exactly at the center of the circle with radius $R_{c}$ and a tangent at {T}. However, for most of the practical applications, the reference paths to follow are curves with slowly varying curvature, this problem is unlikely to occur.

Following an approach similar to that in Section 6.2, the most suitable choice for the desired heading $\psi_{d}$ will be

(144) $$\begin{array}{cc} \psi_{d} & =\sin^{-1}\left\{\sigma_{e}\left(-\frac{K_{1}e}{U}\right)\right\}+\theta_{c}. \end{array}$$

In order to follow a circumference, we compute at each instant the tangent to the path and act as if we were following a straight line. The algorithm applies to the case of straight lines and yields automatic compensation of the effect of unknown but constant currents. The methodology does not go through for the case of general paths even if we restrict ourselves to constant currents. Interestingly enough, as far as we know, this combined problem has not been solved yet. In the paper, we proposed a simple extension of the method to follow arcs of circumference that showed acceptable performance (small steady-state track error) in simulations and real test with the Medusa vehicle (figures are explained in next section); however, a general theoretical result is not available at this point [45].

## 8. Implementation and Field Test Results

**Figure 13:**
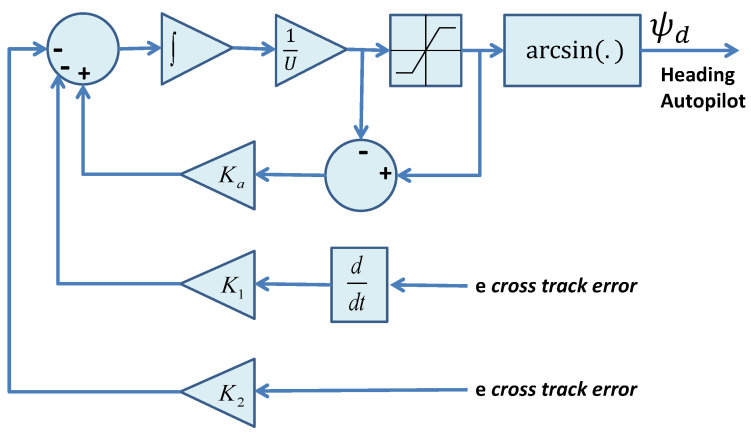
Implementation of the path-following algorithm using an anti-windup technique scheme that includes the so-called D-methodology in [46].

The previous sections described the rationale and provided the mathematical machinery required for the study of a path-following controller for marine vehicles that relies on a two-scale inner-outer loop architecture. This approach effectively decouples the design of the inner and outer control loops, the combination of the two being studied at a later stage. The tools derived borrow extensively from nonlinear control theory and make use of the ISS and IOS characterization of dynamical systems. In this context, the analysis of the combined inner-outer loop structure is done using an appropriate small gain theorem [29]. For inner-loop controller design, the technique described in Section 4 was used, yielding a simple proportional and derivative control law that is pervasive in heading autopilots. In what concerns outer-loop design and stability analysis, despite the apparent complexity of the methodology adopted, the resulting outer-loop controller lends itself to the simple implementation structure shown in Equations (98) and (99) and depicted in Figure 13, where an anti-windup scheme is implemented using the so-called *D-methodology* introduced in [46]. Clearly, the implementation of the outer-loop controller does not require intensive computational power. We recall that the gains $K_{1}$ and $K_{2}$ can easily be computed by solving the characteristic Equation (96) for a choice of natural frequency $\omega_{n}$, with $K_{1}=2\xi \omega_{n}$ and $K_{2}=\omega_{n}^{2}$, where ξ is the damping factor.

**Figure 14:**
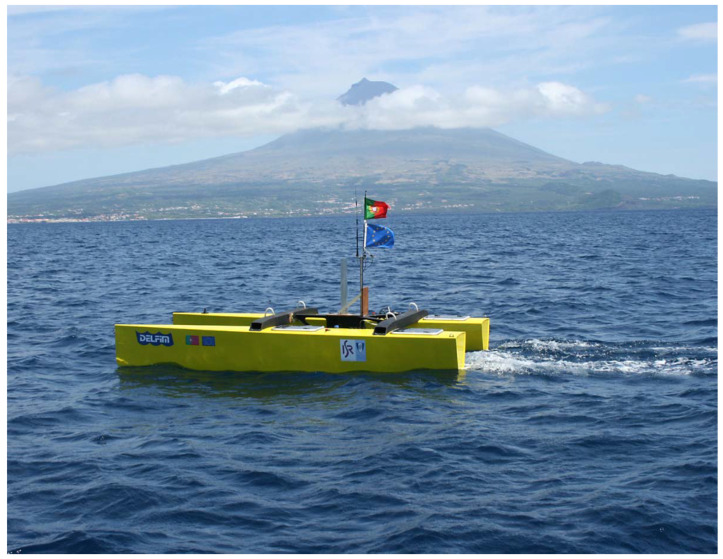
The DELFIM${}_{x}$ ASV.

**Figure 15:**
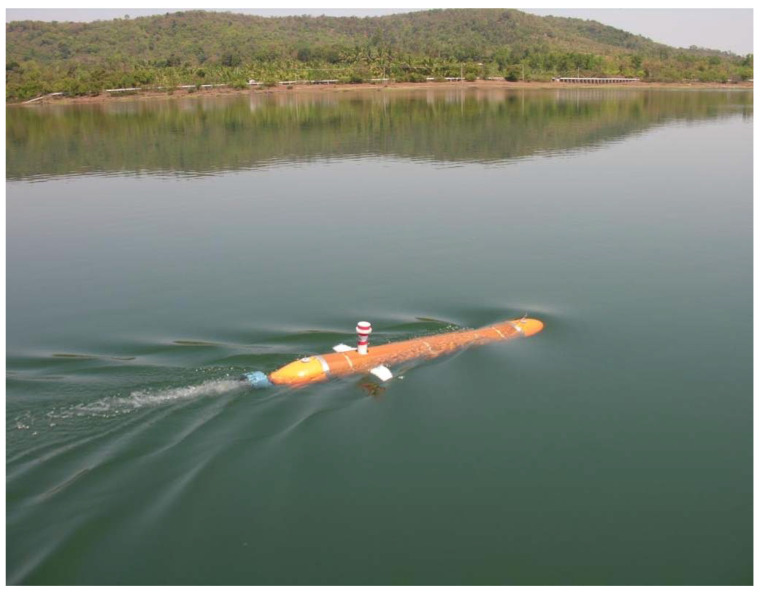
The MAYA AUV.

The algorithm for path following described was implemented and fully tested with success in three types of vehicles: the $DELFIM_{x}$ ASV, the MAYA AUV, and several vehicles of the MEDUSA class. The first is an autonomous surface vehicle that is the property of the Instituto Superior Tecnico, Lisbon, Portugal (see Figure 14). The second is an autonomous underwater vehicle (see Figure 15) described in Section 3.3. Implementation issues and results of tests carried out with the MAYA AUV are briefly discussed in [45]. The algorithm is also an part of the several MEDUSA class vehicles developed by IST, Lisbon. The results are shown for one of the MEDUSA class marine vehicle described in Section 3.2.

Prior to testing the path-following algorithm on the $DELFIM_{x}$ ASV, simulations were done with a full nonlinear model of the vessel. The outer-loop controller parameters were tuned based on the bandwidth of the linearized equations of motion of the vessel about 1.6 m/s. We call attention to the fact that we did not measure the ocean current during the sea trials of $DELFIM_{x}$. However, an estimate was obtained using the difference between the heading and course angles of the vehicle along the straight line components of the path. The estimated current of 0.2 m/s with direction from southwest to northeast was introduced in the simulation to allow for a fair comparison of real and simulated data.

**Figure 16:**
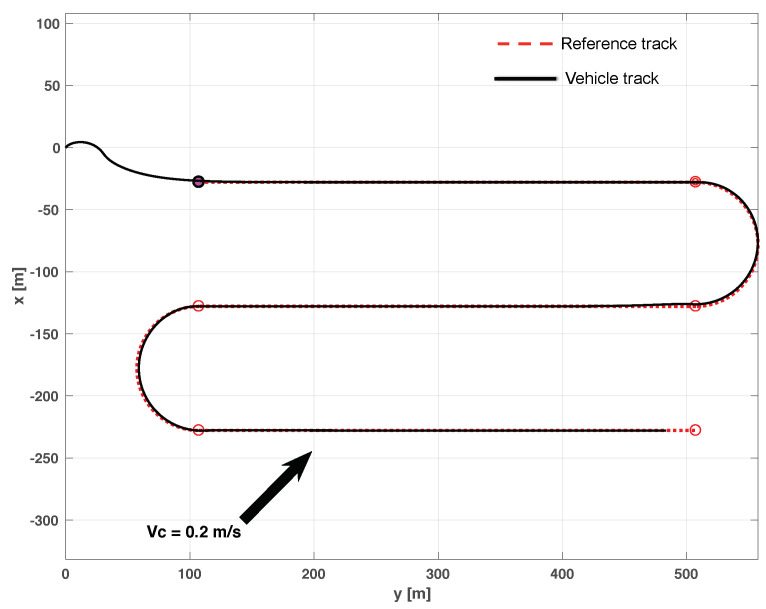
Simulated lawnmowing maneuver of the $DELFIM_{x}$ vehicle in the presence of ocean currents.

**Figure 17:**
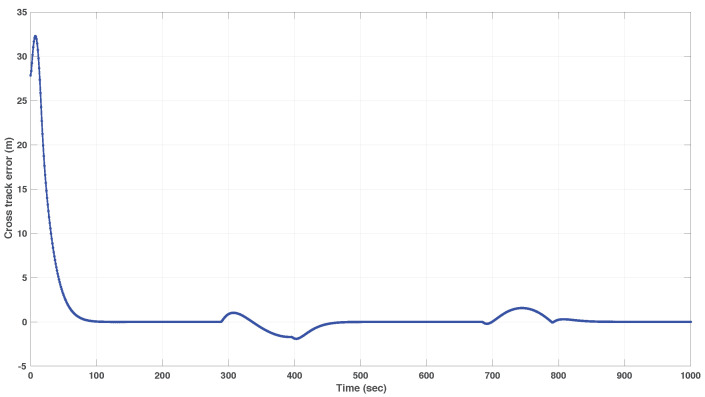
Cross-track error for the simulated track.

**Figure 18:**
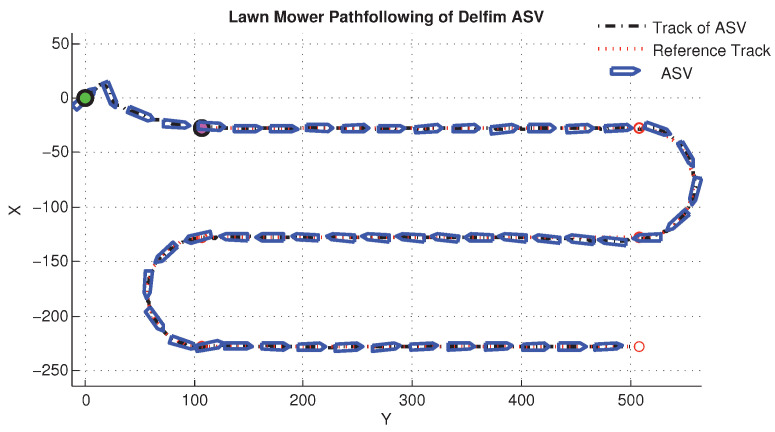
${\mathrm{Delfim}}_{x}$ performing a lawn-mowing maneuver in the Azores, PT.

**Figure 19:**
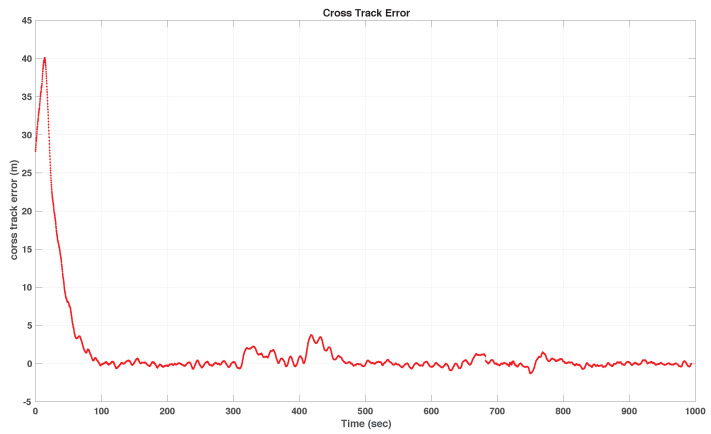
${\mathrm{Delfim}}_{x}$ cross-track error during the real mission.

We include both the results of simulations and actual tests at sea. Namely, Figure 16 and Figure 17 show the results of simulations of a lawn-mowing maneuver for the ASV. Figure 16 illustrates the complete maneuver, whereas Figure 17 shows the cross error observed. The corresponding plots for real tests are shown in Figure 18 and Figure 19, respectively. Clearly, the results of simulations and the real data are very similar, thus confirming the adequacy of the new method developed for path following. Notice in particular how both in simulated and related data the cross-track error converges to approximately zero over similar portions of the path (straight line segments), in the presence of a constant current. The variation in the cross-track error after the convergence at 300 s and 700 s are due to the transition from straight line to the arc and vice versa which is reflected in both simulations and the real tests. Notice that the heading of the vehicle (represented by a the symbol of ASV with the triangular-shaped head (see legend in Figure 18)) is different from course of the vehicle. This shows that the algorithm has learned the ocean currents and adjusted the heading accordingly.

**Figure 20:**
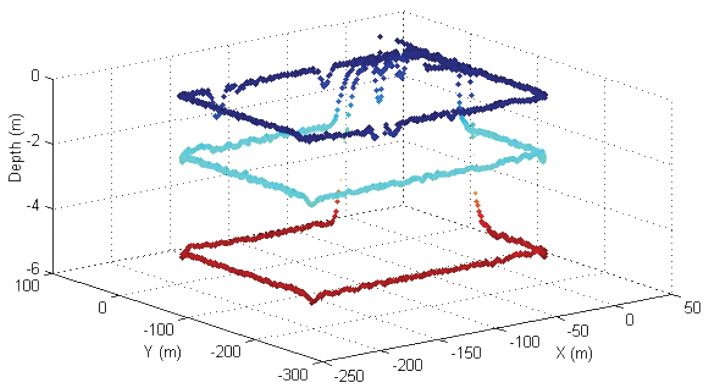
Square mission of MAYA at surface, 3 m and 5 m depth at Supa Dam, India.

Another test was conducted using the MAYA AUV at a lake to map chlorophyll at three different depths. The path-following algorithm used for these tests shows how the AUV was able to follow a path in a real environment independent of the depth control. The results are shown in Figure 20.

**Figure 21:**
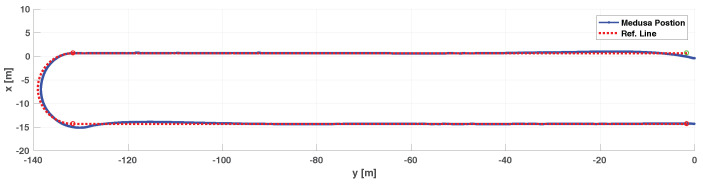
Medusa Vehicle performing lawnmower at Expo Site, Lisbon, Portugal.

**Figure 22:**
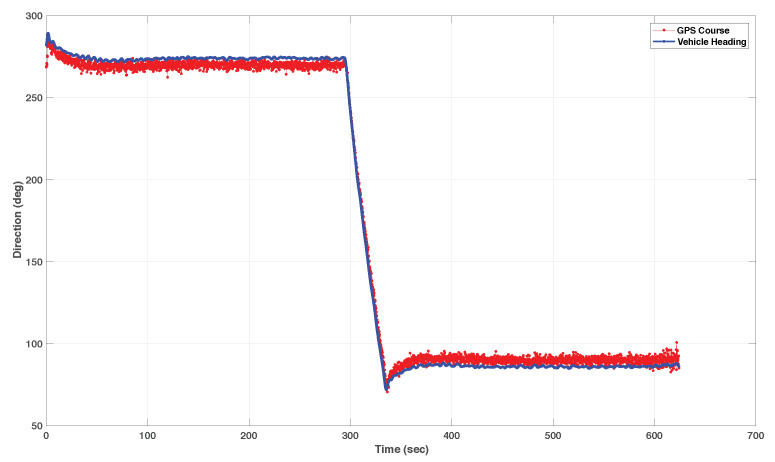
Heading and Course of the Medusa Vehicle Showing the effect of ocean currents.

The efficacy of the path following for straight lines was also shown during a real test with a Medusa class vehicle. The vehicle’s GPS track and the reference path for the test performed are shown in Figure 21. It can be inferred from the fact that the course angle available from GPS and the vehicle angles were not the same that the vehicle was under the influence of an ocean current, see Figure 22. This figure shows clearly the role of the integrator to “learn” the constant ocean current and offset the heading angle accordingly.

**Figure 23:**
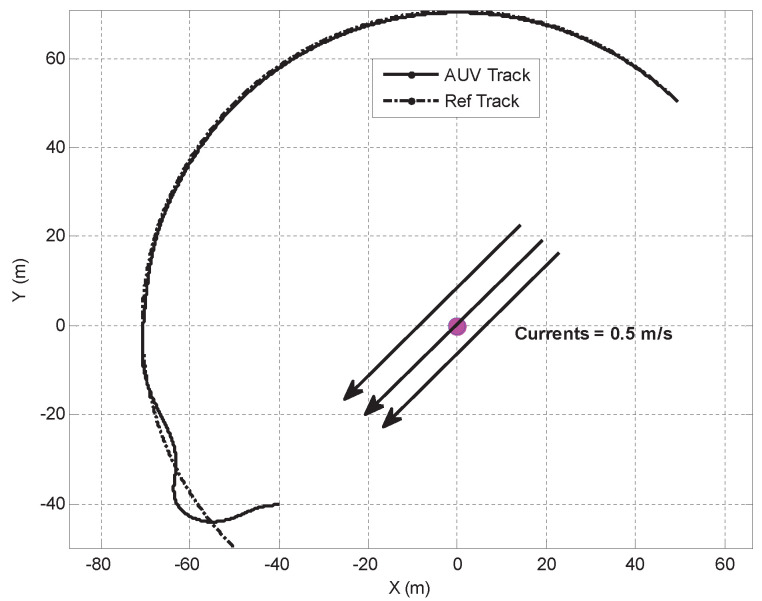
Simulation result of arc following.

**Figure 24:**
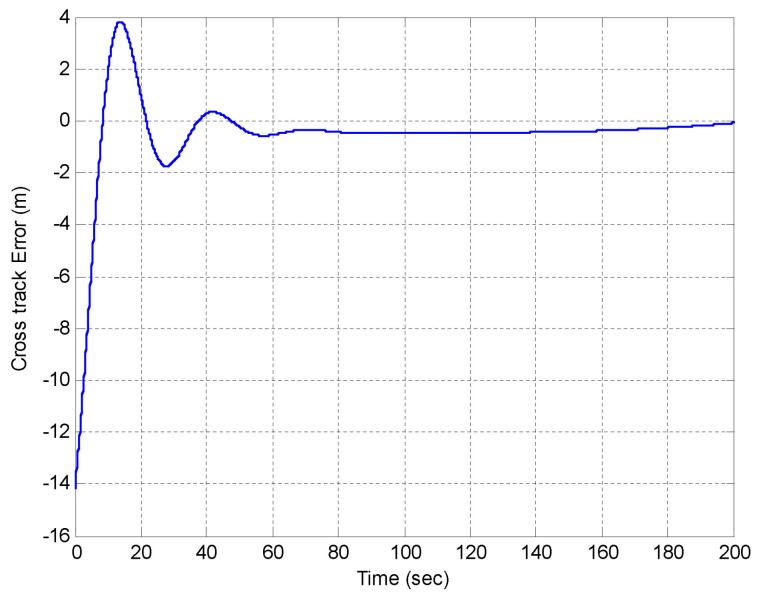
Evolution of cross-track error during arc following.

The simulation results in Figure 23 and Figure 24 illustrate the case where the MAYA vehicle is requested to follow a segment of a circular path. The results show that in the presence of currents, the vehicle follows the arc with an error (i.e., the cross-track error will not converge to zero but to a neighborhood of zero). The convergence of the cross-track error to a neighborhood of zero along segments of arc is also captured in the sea tests performed by $DELFIM_{x}$ and MEDUSA vehicles, as shown in Figure 18 and Figure 21.

## 9. Conclusions and Future Work

This paper introduced an inner-outer control structure for marine vehicle path following in 2D, with due account for the vehicle dynamics and ocean currents. The structure is simple to implement and provides system designers a convenient way of tuning the outer-loop control law parameters as functions of a “bandwidth-like” characterization of the inner loop. Stability of the complete path-following control system was proven for straight paths, by resorting to nonlinear control theoretical tools that borrow from input-to-output stability concepts and a related small gain theorem. The efficacy of the inner-outer control structure adopted was shown during the rigorous tests with AUVs and ASVs at sea. These algorithms are now integral part of many autonomous marine vehicles used at NIO and IST. Moreover, the problem of cooperative control and navigation works on the assumption that the single vehicle is able to follow a desired path (without any temporal constraints).

The applications of this strategy include: (i) single-vehicle path following for a number of missions that include environmental surveying, seabed habitat mapping, and critical infrastructures security, and (ii) cooperative path following, which aims to steer a number of vehicles along pre-determined paths in a synchronized manner, with a view to overcoming the limitations imposed by the use of a single vehicle, effectively allowing for ocean exploration at unprecedented temporal and spatial scales. The method is easily extended to fully actuated or overly actuated vehicles where, besides having the center of mass of the vehicle follow a desired path, it is also required for the vehicle to track an arbitrary heading reference (that is, complete pose control). An obvious desired extension (pointed out before) is to derive a path-following controller capable of ensuring precise path following of general paths in the presence of constant currents. We conjecture that some form of an internal model principle should be used, which provides a good ground for future extension of the present work, see, for example, Refs. [47,48,49] and the references therein.

We also remark that we have addressed explicitly the effect of unknown but constant currents and showed how the path-following control law adopted allows for the rejection of this type of disturbance. We did not address the impact of waves, which cause first-order (oscillating) and second-order (drift) effects. We conjecture that the influence of waves may be studied by modeling (as is customary in the literature) their effect as a bounded output disturbance $d_{w}$, and characterizing the closed-loop operator with input $d_{w}$ and output *e* (cross-track error) in terms of its input-output characteristics (IOS analysis). 

## Figures and Tables

**Figure 8:**
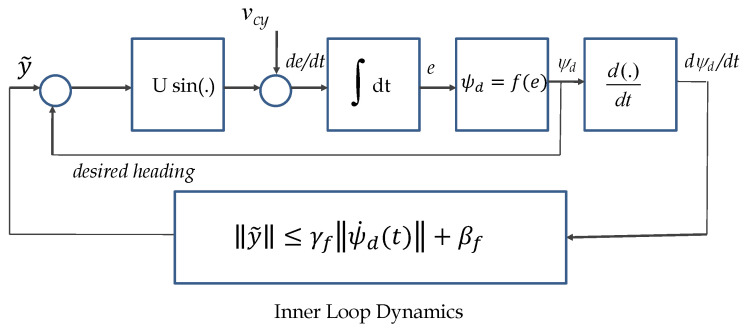
IOS characterization of inner-outer loop.

**Figure 9:**
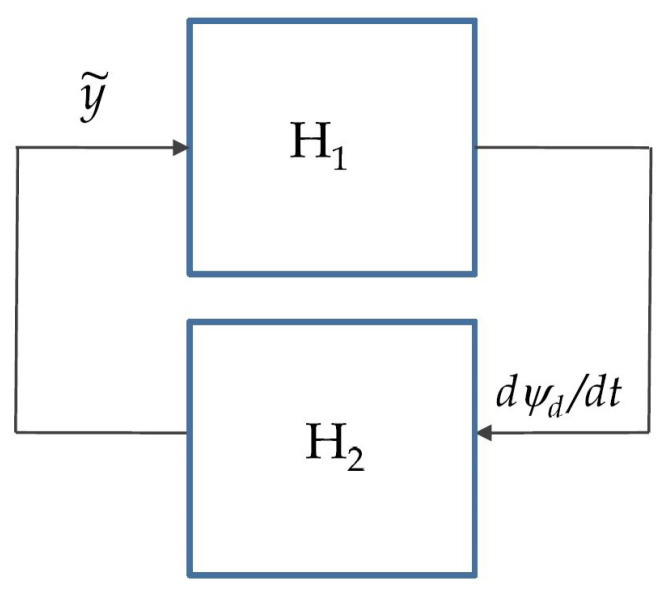
General feedback interconnection.

**Table 1:** Parameters to design outer loop.

Parameters	Value
Speed U	1 m/s
y-component of current $V_{yc}$	0.1 m/s
saturation $e_{s}$	0.8
**Parameters used for**	**with $K_{2}=\omega_{n}^{2}$**
**outer loop design**	**and $K_{1}=2\xi \omega_{n}$**
damping factor ξ	0.8
δ	0.083
θ	0.99

## Data Availability

Not applicable.

## Appendix A. Vehicle Parameters

**Table A1:** Mass, inertia and hydrodynamics coefficients for a MEDUSA vehicle.

m = 17 kg	$I_{z}=1$ kg·m${}^{2}$	
$X_{\dot{u}}=-20$ kg	$Y_{\dot{v}}=-30$ kg	$N_{\dot{r}}=-8.69$ kg·m${}^{2}$
$X_{u}=-0.2$ kg/s	$Y_{v}=-50$ kg/s	$N_{r}=-4.14$ kg·m${}^{2}$/s
$X_{|u|u}=-25$ kg/m	$Y_{|v|v}=-0.01$ kg/m	$N_{|r|r}=-6.23$ kg·m${}^{2}$

**Table A2:** Vehicle parameters of MAYA AUV.

Physical Parameters	
Vehicle Speed $\left(u_{0}\right)$	: 1.2 m/s
Reynolds Number	: $1.6692\times 10^{6}$
Sea Water Density $\left(\rho \right)$	: 1025 kg/m${}^{3}$
**Vehicle Parameters**	
Length $\left(L_{pp}\right)$	: 1.8 m
Center of mass $\left(z_{g}\right)$	: $0.52\times 10^{-2}$ m (w.r.t body geometric axis)
Center of Buoyancy $\left(z_{b}\right)$	: $-0.172\times 10^{-2}$ m (w.r.t body geometric axis)
Weight (W)	: 53 × 9.8 kgf
Buoyancy (B)	: 53.4 × 9.8 kgf
**Hydrodynamic Parameters**	
*Moments Coeff.*	*Force Coeff.*
$N_{r}$ = −41.4397 kg·m${}^{2}$/s	$Y_{r}$ = 70.4195 kg·m/s
$N_{\delta }$ = −33.8563 kg·m${}^{2}$/s${}^{2}$	$Y_{\delta }$ = 96.3191 kg·m/s${}^{2}$
$N_{v}$ = −29.8727 kg·m/s	$Y_{v}$ = −204.698 kg/s
$N_{\dot{r}}$ = −41.4397 kg·m${}^{2}$	$Y_{\dot{r}}$ = 70.4195 kg·m
$N_{\dot{v}}$ = −29.8727 kg·m	$Y_{\dot{v}}$ = −204.698 kg
